# Survival-Inferred Fragility Index in Phase III Colorectal Cancer Trials: A Systematic Review

**DOI:** 10.3390/cancers18203394

**Published:** 2026-10-20

**Authors:** Lanwei Guo, Jiani Yuan, Xuejing Zhang, Lin Cai, Xinxin Yan, Huamei Luan, Chenxin Zhu, Yan Zheng, Yanyan Liu, Huiyao Huang

**Affiliations:** 1 Department of Clinical Research Management, The Affiliated Cancer Hospital of Zhengzhou University and Henan Cancer Hospital, Zhengzhou 450008, China; 2 Department of Epidemiology, School of Public Health, Zhengzhou University, Zhengzhou 450001, China; 3 Clinical Trials Center, National Cancer Center, National Clinical Research Center for Cancer, Cancer Hospital, Chinese Academy of Medical Sciences, Peking Union Medical College, 17 South Panjiayuan Lane, Beijing 100021, China; 4 IRB Office, National Cancer Center, National Clinical Research Center for Cancer, Cancer Hospital, Shenzhen Hospital, Chinese Academy of Medical Sciences, Peking Union Medical College, No. 113, Baohe Avenue, Shenzhen 518116, China

**Keywords:** survival-inferred fragility index, randomized controlled trials, colorectal cancer

## Abstract

Background: Conventional measures such as *p*-values and hazard ratios (*HRs*) do not directly quantify the sensitivity of time-to-event outcomes to small changes in individual outcomes. The Survival-Inferred Fragility Index (SIFI) evaluates the minimum number of survival-outcome changes required to reverse trial conclusions and provides complementary information on result robustness. Methods: This systematic review (PROSPERO: CRD420251182325) identified phase III colorectal cancer randomized controlled trials (RCTs) published from 2015 to 2025 via PubMed, Embase, and Cochrane. SIFI was calculated using Bomze’s framework with the “flip best” strategy. The fragility quotient (FQ) and the relationship between SIFI and withdrawals or losses to follow-up (WCLFU) were assessed. Analyses used R version 4.5.1. Results: SIFI was calculated for 50 phase III colorectal cancer RCTs, including 9 three-arm trials, resulting in 59 pairwise comparisons. Among positive results, median SIFI values for colorectal, colon, and rectal cancer were 6.0 (interquartile range [IQR]: 2.0 to 11.0), 19.0 (IQR: 15.5 to 22.5), and 4.0 (IQR: 4.0 to 4.0). Higher SIFI was observed in trials with *HR* < 0.75 than in those with *HR* 0.75–0.94 (median SIFI: 9.0 vs. 1.0; *p* = 0.035) and in trials with power > 85% than in those with power ≤ 85% (median SIFI: 8.5 vs. 2.0; *p* = 0.028). For negative results, median SIFI values were −4.5 (IQR: −7.2 to −3.0), −30.5 (IQR: −43.5 to −18.0), and −10.5 (IQR: −11.0 to −5.5). Greater absolute SIFI values were associated with adjuvant therapy, sample size ≥ 500, disease-free survival as the primary endpoint, and *HR* ≥ 0.95 (all *p* ≤ 0.005). Overall, 44.07% of pairwise comparisons had an absolute SIFI < 5. Conclusions: SIFI shows some phase III colorectal cancer RCT conclusions were sensitive to small outcome changes. It complements conventional statistics by illuminating result robustness.

## Simple Summary

Clinical trials can show whether a treatment improves outcomes, but conventional statistical measures do not always indicate how robust these findings are to small changes in patient outcomes. The Survival-Inferred Fragility Index is a measure that estimates how many individual survival outcomes would need to change to reverse a trial’s original conclusion. We systematically reviewed phase III randomized controlled trials of colorectal cancer published from 2015 to 2025 and applied this measure to 59 treatment comparisons from 50 trials. We found that some statistically significant and nonsignificant results could be changed by a relatively small number of outcome changes. Trials with larger treatment effects and stronger statistical power generally required more outcome changes to alter their original conclusions. These findings suggest that this measure can provide complementary information to conventional statistical results and help researchers better understand the robustness of evidence from colorectal cancer clinical trials.

## 1. Introduction

Colorectal cancer is a malignant tumor that originates in the mucosal epithelium of the colon or rectum. Locally, it can invade from the submucosal layer into the muscularis propria, serosa, and adjacent organs, and most commonly spreads to the liver, lungs, and peritoneum via lymphatic, hematogenous, and peritoneal routes [1,2,3]. According to data from GLOBOCAN 2024 [4], colorectal cancer is the third most commonly diagnosed cancer worldwide, with approximately 1.1 million new cases in men and 0.9 million in women. In terms of cancer mortality, colorectal cancer ranks as the second leading cause of cancer death among men (approximately 500,000 deaths) and the third leading cause among women (approximately 400,000 deaths).

Randomized controlled trials (RCTs) represent the cornerstone of oncology, with their findings traditionally inferred using *p*-values, hazard ratios (*HRs*), and their corresponding confidence intervals (*CIs*). However, the reliability of both metrics has come under increasing scrutiny in recent years. Rubinstein conducted a systematic review and meta-analysis of 556 published oncology abstracts with marginally significant results (*p*-values between 0.01 and 0.10) and found that the results of oncology RCTs exhibit a marked tendency toward “over-affirmation,” with nearly 40% of the abstracts failing to acknowledge uncertainty in the results—a phenomenon observed in clinical trials for non-small cell lung cancer, breast cancer, and other malignancies [5]. On the other hand, when the proportional hazards assumption underlying the Cox model is violated over time, the reliability of conventional hazard ratio estimates and their interpretation may be reduced [6].

To address these shortcomings, Walsh proposed the Fragility Index (FI) in 2014, transforming “statistical significance” into a more intuitive measure of robustness. FI is calculated based on a 2 × 2 contingency table, using the distribution of raw outcomes as a baseline. In groups with a small number of events, one non-event is progressively reclassified as an event, and a two-sided Fisher’s exact test is repeated until the *p*-value reaches or exceeds 0.05. The FI is defined as the minimum number of outcome status changes required to turn a study result from statistically significant to non-significant. A higher FI indicates that more changes in patient outcomes are required to alter the statistical conclusion, suggesting that the study results are more robust [7]. However, the calculation of FI relies on an average “virtual patient” and ignores the extreme cases that play a key role in the shape of the curve—namely, the “best survivors” at the far right of the survival curve and the “worst survivors” at the far left. In response, Bomze further expanded the concept of FI and proposed the Survival Fragility Index (SIFI), defined as the minimum number of “best survivors” (i.e., patients with the longest follow-up duration, regardless of whether an event occurred or data were missing) that must be reallocated from the experimental group to the control group in order to eliminate statistical significance [8]. Because SIFI incorporates individual-level survival information and the timing of events and censoring, it provides a survival-specific framework for evaluating the statistical robustness of time-to-event outcomes. Previous studies on the FI in RCTs of colorectal cancer have primarily focused on the evaluation of positive results and have largely employed traditional FI calculation frameworks; systematic assessments of the stability of statistical conclusions in negative trials remain relatively limited.

The purpose of this systematic review was to systematically evaluate the statistical robustness of positive and negative colorectal cancer RCTs using SIFI, thereby addressing the aforementioned limitations. For positive trials, SIFI is used to evaluate the robustness of statistically significant results; for negative trials, reverse SIFI is used to measure the sensitivity of negative results to changes in the underlying event. This bidirectional evaluation framework not only identifies statistically significant but statistically unstable study results but also helps assess the sensitivity of potential treatment effects to changes in events in negative trials, providing a new perspective for future clinical trial design and interpretation of results.

## 2. Methods

### 2.1. Study Design

This systematic review was conducted in accordance with the “Preferred Reporting Items for Systematic Reviews and Meta-Analyses” (PRISMA) guidelines and has been prospectively registered on the PROSPERO (CRD420251182325). The completed PRISMA checklist is provided as Table S9. We systematically searched three databases—PubMed, Embase, and the Cochrane Library—to identify phase III RCTs published between 2015 and 2025 across 10 top-tier international medical and oncology journals: The New England Journal of Medicine, The Lancet, JAMA, BMJ, Journal of Clinical Oncology, The Lancet Oncology, Annals of Oncology, British Journal of Cancer, European Journal of Cancer, and JAMA Oncology. Detailed search strategies are presented in Tables S2–S4. For the SIFI analysis of colorectal cancer, this study included only two- or three-arm parallel-group controlled trials with time-to-event outcomes as the primary endpoint or key secondary endpoints. Exclusion criteria comprised non-original research, retrospective or post-hoc subgroup analyses, non-pharmacological interventions, studies focusing solely on biosimilars or drug-device combinations, trials not involving colorectal cancer, and publications ineligible due to other reasons such as duplicate publications, non-randomized controlled study designs, or non-oncology studies. When multiple publications originating from the same trial were identified, only the most recent version was included. Detailed inclusion and exclusion criteria are illustrated in Figure S1.

Because this systematic review represents a secondary analysis of previously published aggregate data, with all information derived from publicly accessible literature, institutional review board approval was not required.

### 2.2. Data Extraction

Data extraction was performed independently by two investigators (JNY and HML). Any disagreements were resolved through discussion, with unresolved discrepancies adjudicated by a third investigator (LWG). The extracted variables encompassed trial design characteristics and key outcome measures, including cancer type (colon, rectal, or colorectal cancer), intervention, trial name/NCT (National Clinical Trial) number, publication year, source of funding (investigator-initiated trial [IIT] vs. industry-sponsored trial [IST]), total sample size, withdrawal of consent or lost to follow-up (WCLFU, defined as the total number of patients in the experimental group who withdrew consent or were lost to follow-up), statistical power, median follow-up time, primary endpoint, *HR* (hazard ratio), and the corresponding *p*-values.

To facilitate the calculation of SIFI, we reconstructed individual patient-level survival times and censoring statuses through the following steps. First, Kaplan–Meier curves of survival data were extracted from the 50 included colorectal cancer trials using DigitizeIt software (version 2.6.6). The published Kaplan–Meier curves were digitized by calibrating the axes and extracting the coordinates of the survival curves at multiple time points. Where available, the numbers at risk and other reported survival information were also recorded from the original publications. Of these, 9 were three-arm trials, resulting in 59 pairwise comparisons for subsequent SIFI analyses. Subsequently, individual patient-level survival times and censoring indicators were reconstructed in Stata (version 18.0) using the algorithm described by Wei and Royston [9]. The reconstructed survival data were then used for subsequent SIFI calculations. To assess the consistency between the reconstructed and originally reported results, the original and reconstructed *HRs* and *p*-values were compared using intraclass correlation coefficients (ICCs) and mean differences with 95% *CI*.

### 2.3. Statistical Analysis

The calculation of the SIFI in this study adopted the survival inference fragility index framework proposed by Bomze, which encompasses four simulation strategies: “flip best”, “flip worst”, “clone best”, and “clone worst” [8]. This approach calculates the minimum number of patient status changes required to alter a trial’s statistical conclusion by reallocating or cloning patients with extreme survival outcomes. Specifically, the “flip best” strategy iteratively reallocates the best survivors (i.e., patients with the longest survival times) from the intervention group to the control group, repeatedly executing two-sided log-rank tests until statistical significance is lost (*p* ≥ 0.05); the required number of reallocated patients is defined as the SIFI. Conversely, a negative SIFI is calculated in a similar iterative manner by reallocating the best survivors from the control group to the intervention group. A negative SIFI indicates the direction of change. Its absolute value represents the number of patient outcomes needed to alter the original conclusion. Larger absolute SIFI values indicate greater statistical robustness. The “flip worst” strategy involves reallocating the worst survivors (i.e., patients experiencing events earliest) from the experimental group to the control group. The “clone best” strategy clones the best survivors from the experimental group into the control group, whereas the “clone worst” strategy clones the worst survivors from the control group into the experimental group. In this study, “flip best” was adopted as the primary method for SIFI calculation. The relevant analytical code was adapted from the publicly available scripts provided by Bomze [8].

To provide a normalized measure of SIFI relative to trial enrollment, we further calculated the fragility quotient (FQ), defined as the ratio of SIFI to the total sample size. A lower FQ indicates that changes in survival outcomes among a smaller proportion of patients can influence the study’s conclusions, suggesting weaker result stability. Since there is currently no widely accepted standard for evaluating SIFI, we further examined the relationship between SIFI and WCLFU. When WCLFU exceeded the absolute value of SIFI, the study results were considered potentially sensitive to loss to follow-up. Firth penalized multivariable logistic regression analysis was performed to explore trial characteristics associated with WCLFU exceeding the absolute value of SIFI.

Continuous variables are presented as medians (interquartile range), and categorical variables are presented as frequencies (percentages). Survival curves were estimated using the “prodlim” package, and comparisons between groups were performed using the two-sided unstratified log-rank test. Depending on the distribution of the variables, either Pearson or Spearman correlation analysis was selected to evaluate the relationship between study characteristics and SIFI. These analyses were considered exploratory, and no adjustment for multiple comparisons was performed. Simulated survival datasets were generated using the “survsim” package to explore the range of SIFI values under different conditions. All statistical tests were two-sided; a *p*-value < 0.05 was considered statistically significant. All analyses were performed using R (version 4.5.1).

## 3. Results

### 3.1. Characteristics of the Included Trials

**Figure 1:**
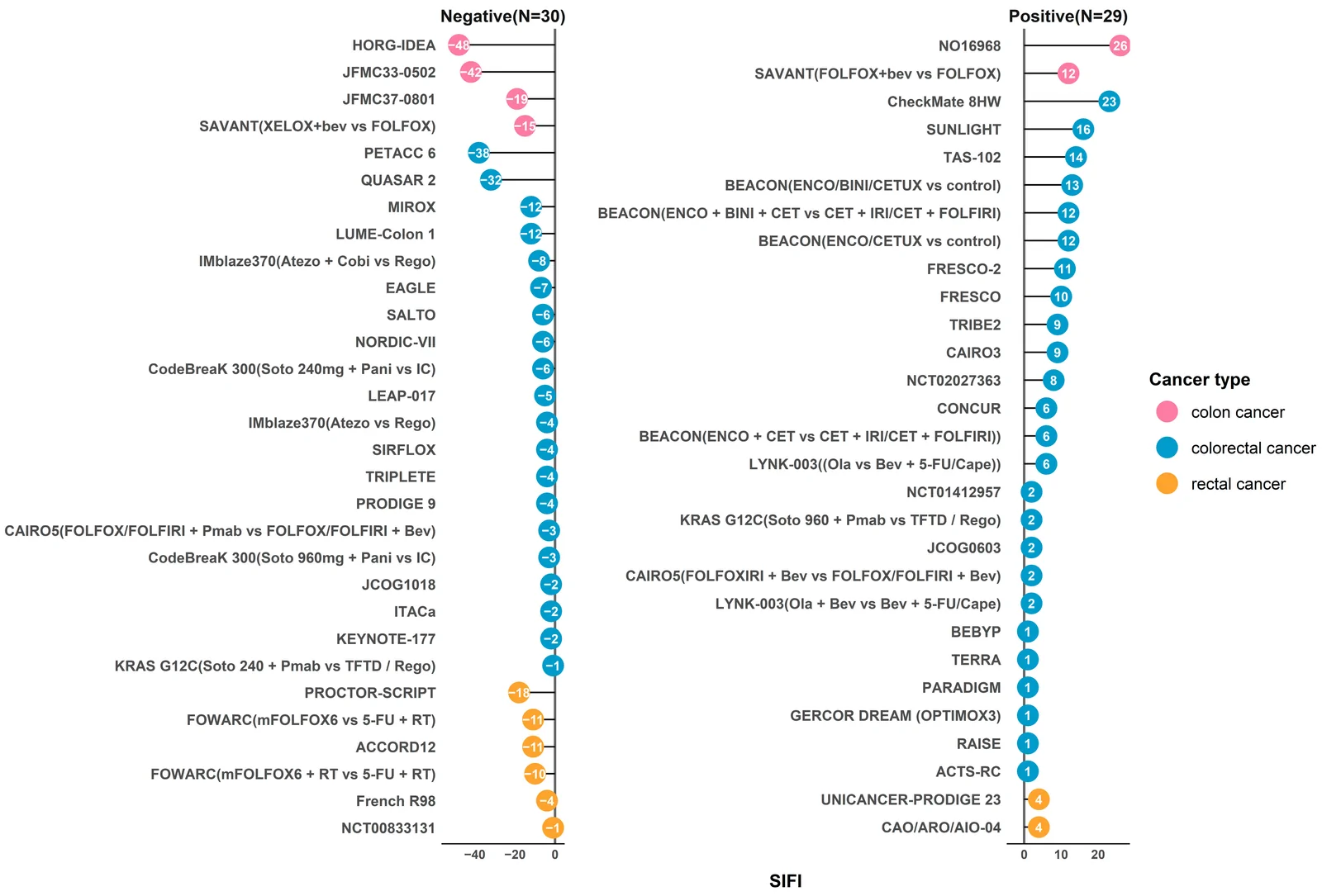
Survival-Inferred Fragility Index (SIFI) by cancer type.

In accordance with the PRISMA guidelines, this study ultimately included 50 Phase III RCTs (including 9 three-arm studies) (Table S1) [10,11,12,13,14,15,16,17,18,19,20,21,22,23,24,25,26,27,28,29,30,31,32,33,34,35,36,37,38,39,40,41,42,43,44,45,46,47,48,49,50,51,52,53,54,55,56,57,58,59]. Trials related to colorectal cancer accounted for the largest proportion (*n* = 45) [11,13,14,15,16,17,19,21,23,25,26,27,29,30,32,35,37,38,42,44,45,46,47,48,49,51,52,53,54,58,59], followed by rectal cancer (*n* = 8) [18,34,36,40,41,43], and finally colon cancer (*n* = 6) [20,22,24,28]. The SIFI for the included trials are shown in Figure 1.

A total of 29 positive pairwise comparisons were included; colorectal cancer was the most common cancer type (*n* = 25, 86.2%), with 2 cases each of colon cancer and rectal cancer (6.9%). The median SIFI for the three groups were 6.0 (IQR: 2.0 to 11.0), 19.0 (IQR: 15.5 to 22.5), and 4.0 (IQR: 4.0 to 4.0), respectively. Most positive trials were IST (65.5%), with OS as the primary endpoint (44.8%) and an *HR* < 0.75 (62.1%); targeted therapy was the most common drug class (41.4%) (Table 1).

A total of 30 negative pairwise comparisons were included, among which colorectal cancer accounted for the highest proportion (*n* = 20, 66.7%), followed by rectal cancer (*n* = 6, 20.0%) and colon cancer (*n* = 4, 13.3%). The corresponding median SIFIs for these three types were −4.5 (IQR: −7.2 to −3.0), −10.5 (IQR: −11.0 to −5.5), and −30.5 (IQR: −43.5 to −18.0), respectively. Among the negative trials, the vast majority (96.7%) did not employ a double-blind design, 66.7% had a sample size of <500, 90% utilized active controls, and chemotherapy combined with targeted therapy was the most common drug category (*n* = 9, 30%) (Table 2).

### 3.2. SIFI by Trial Characteristics

Subgroup analysis based on test characteristics revealed that SIFI exhibited different distribution patterns between positive and negative results. In tests with positive results, SIFI exploratorily associated with the distributions of Power and *HR*. Among these, the association between *HR* and SIFI showed a more pronounced difference in effect size. The median SIFI was 9.0 (IQR: 4.5 to 12.0) in the *HR* < 0.75 group, compared with 1.0 (IQR: 1.0 to 2.5) in the *HR* 0.75–0.94 group (*p* = 0.035). Differences in power across groups were also statistically significant (*p* = 0.028), but the effect size was relatively small: the median SIFI in the power > 85% group was 8.5 (IQR: 2.5 to 13.5), higher than the 2.0 (IQR: 1.0 to 8.0) in the ≤85% group. However, other characteristics—such as cancer type, year of publication, study type, and use of blinding—were not significantly associated with SIFI (Table 1).

Unlike positive results, several trial characteristics among trials with negative results, including cancer type, publication year, sample size, line of therapy, primary endpoint, and *HR*, showed associations with SIFI in the exploratory subgroup analyses. Regarding the primary endpoint, the DFS group had the lowest median SIFI, at −32.0 (IQR: −40.0 to −15.5), compared with the OS group (−6.0 [IQR: −11.0 to −4.2]) and the PFS group (−4.0 [IQR: −6.0 to −2.0]; *p* < 0.001). In terms of sample size, the median SIFI was lower in trials with a total sample size of ≥500 than in those with a sample size of <500 (−17.0 [IQR: −36.5 to −11.2] vs. −4.5 [IQR: −7.2 to −3.0]; *p* = 0.005). When stratified by line of therapy, the adjuvant therapy group had the lowest median SIFI, at −19.0 (IQR: −37.0 to −16.5), compared with the first-line and second-line therapy groups (*p* = 0.005). SIFI values also differed across the *HR* categories (*p* = 0.003). The median SIFI was −1.5 (IQR: −2.2 to −1.0) in the *HR* < 0.75 group, compared with −6.0 (IQR: −11.0 to −4.0) in the *HR* 0.75–0.94 group and −12.0 (IQR: −35.0 to −6.5) in the *HR* ≥ 0.95 group (Table 2).

To further assess the potential impact of WCLFU on the robustness of the trial conclusions in real-world settings, we used whether the number of WCLFU cases exceeded the absolute value of SIFI as the dependent variable and performed Firth penalized logistic regression to explore relevant trial characteristics. Among the 59 pairwise comparisons, 14 were classified as events and 45 as non-events. The model included 14 non-intercept parameters, corresponding to an events-per-parameter ratio of 1.0. None of the examined trial characteristics showed a statistically significant association with the outcome (Table 3). However, the wide confidence intervals indicate substantial uncertainty in the estimates.

### 3.3. Correlation Analysis Between SIFI and Experimental Characteristics

**Figure 2:**
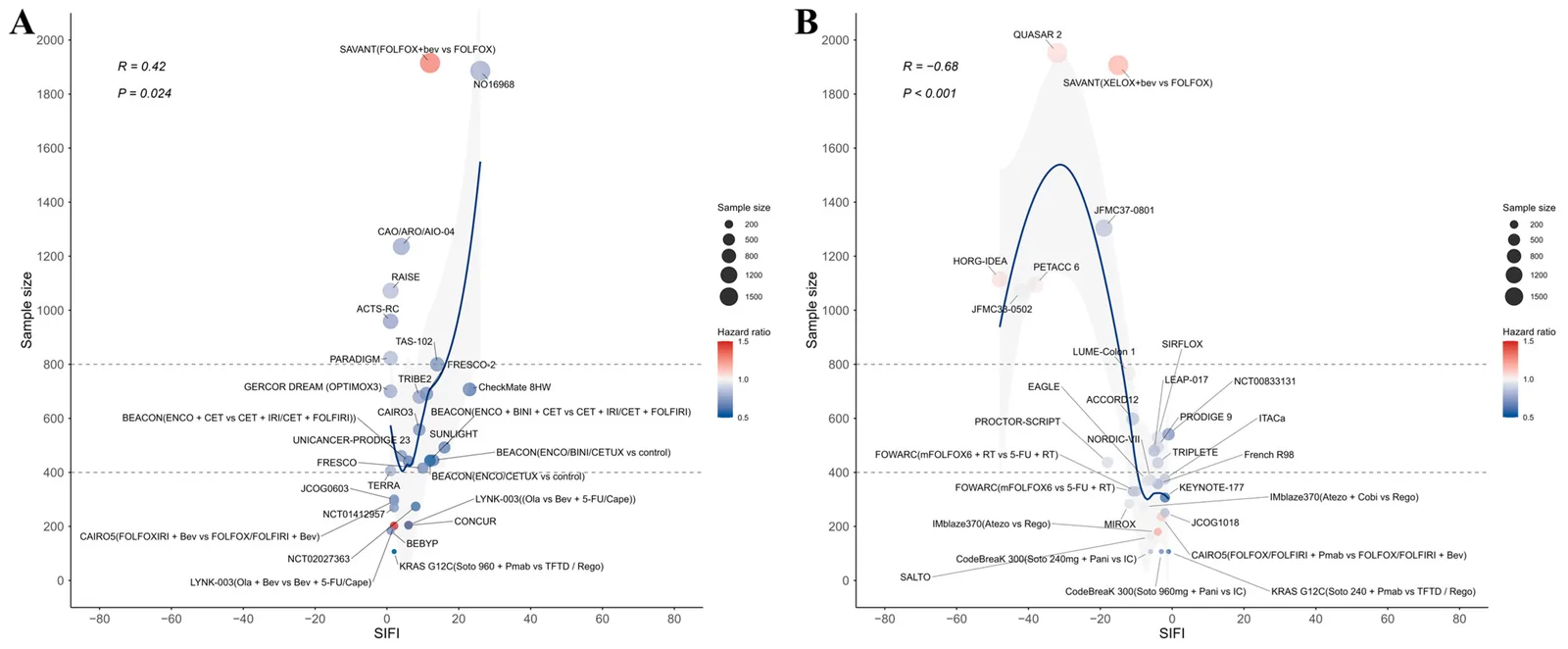
Correlation between Survival-Inferred Fragility Index (SIFI) and sample size in all included colorectal cancer pairwise comparisons. (**A**) Positive results, (**B**) negative results. The gray shaded area represents the 95% confidence interval around the fitted curve. The dashed horizontal lines indicate sample sizes of 400 and 800, respectively, to facilitate visual assessment of the sample size distribution. The solid curve represents the fitted trend between SIFI and sample size.

Stratified by trial results, SIFI was positively correlated with sample size among trials with positive results, with larger trials generally exhibiting higher SIFI values (*R* = 0.42, *p* = 0.024). In contrast, among trials with negative results, SIFI was negatively correlated with sample size (*R* = −0.68, *p* < 0.001), indicating that the absolute value of SIFI progressively increased with increasing sample size (Figure 2).

**Figure 3:**
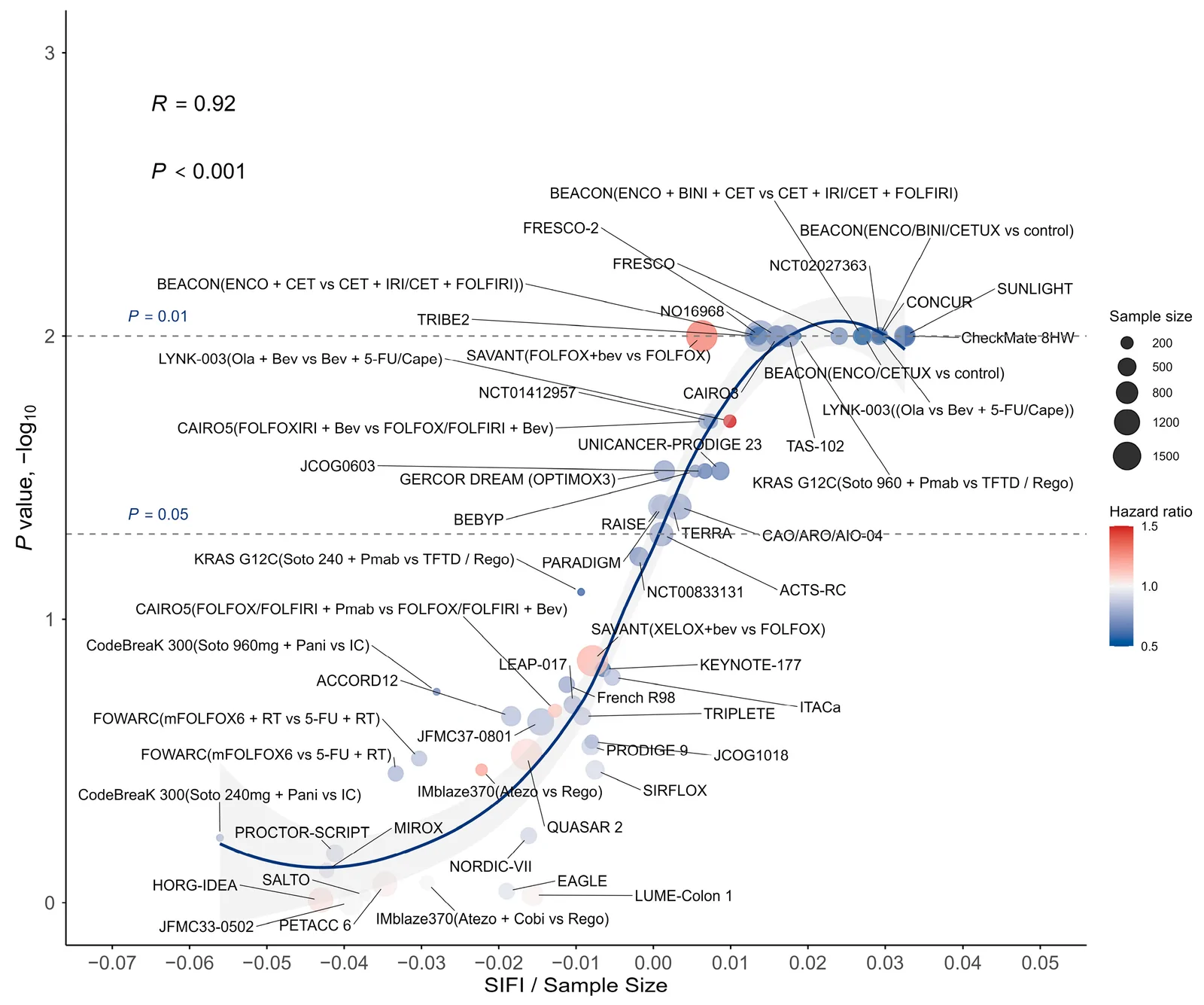
Correlation between Survival-Inferred Fragility Index (SIFI) as a proportion of the sample size and *p*-values in a-logarithmic scale across all included pairwise comparisons. The gray shaded area represents the 95% confidence interval around the fitted curve. The dashed horizontal lines indicate the significance thresholds of *p* = 0.05 and *p* = 0.01, respectively. The solid curve represents the fitted trend between SIFI as a proportion of the sample size and *p*-values on a −log_10_ scale.

Further analysis of the relationship between SIFI and (−log_10_(*p*-value)) revealed a significant positive correlation between the two (Figure S2, ρ = 0.94, *p* < 0.001), indicating a close relationship between statistical evidence and SIFI. Given that SIFI is highly influenced by sample size, we introduced the FQ to normalize SIFI to trial enrollment and reanalyzed the data. The results showed that FQ remained significantly positively correlated with −log_10_(*p*-value) (Figure 3, *R* = 0.92, *p* < 0.001), indicating a close relationship between normalized SIFI and the strength of statistical evidence.

Across all included trials, the absolute value of SIFI was not significantly correlated with median follow-up time (Figure S3, ρ = 0.14, *p* = 0.31). Further stratified analysis by trial outcome revealed no significant correlation between SIFI and power. In positive trials, the two variables showed a trend toward a positive correlation but did not reach statistical significance (ρ = 0.39, *p* = 0.064); no significant correlation was found in negative trials either (ρ = −0.16, *p* = 0.430) (Figure S4).

### 3.4. Sensitivity Analysis

To verify the methodological reliability of the SIFI calculation based on reconstructed survival data, this study assessed the consistency between the originally reported results and the simulated results. The original results and the simulated results showed good agreement in terms of *p*-values and log(*HR*), with ICCs of 0.80 (95% *CI*: 0.68 to 0.88) and 0.86 (95% *CI*: 0.76 to 0.91), respectively (both *p* < 0.001) (Table S5). A detailed comparison of the reported and simulated *HRs* and *p* values is provided in Table S6. These results suggest that methods based on reconstructed survival data can effectively reproduce the statistical characteristics of the original trial.

To assess the impact of different SIFI calculation strategies on the study conclusions, we compared the results obtained using four calculation methods: flip best, flip worst, clone best, and clone worst. As shown in Figure S5, among the four methods, the flip best method yielded the lowest overall absolute SIFI values, corresponding to the smallest number of changes in patient outcomes required to alter the original statistical conclusion. This finding is consistent with the “minimum vulnerability change” principle underlying the SIFI framework. Therefore, the flip best method was selected as the primary analysis method in this study. To further assess whether the main findings were dependent on a specific SIFI calculation strategy, we compared the correlation patterns between SIFI and trial characteristics, including sample size, *HR*, median follow-up time, and statistical power, across the four methods. The results showed similar correlation patterns across the four calculation methods in both positive and negative trials (Figure S6), indicating that the main findings were robust to the choice of SIFI calculation method.

To address the potential non-independence of comparisons from three-arm trials, we excluded these comparisons and repeated the descriptive and correlation analyses for positive and negative results. The findings remained consistent with the primary analysis (Tables S7 and S8; Figures S7 and S8), suggesting that the main results were robust to the exclusion of three-arm trials.

## 4. Discussion

This study calculated the SIFI for 50 phase III RCTs of colorectal cancer, including 9 three-arm trials, resulting in 59 pairwise comparisons for SIFI analyses. The results showed that 44.07% of pairwise comparisons had an absolute SIFI value of less than 5. This finding suggests that a significant proportion of Phase III RCTs of colorectal cancer have fragile survival outcomes, and that statistical significance could potentially be reversed by simply reassigning a small number of “best survivors.”

However, there are currently no widely accepted criteria for assessing the stability of SIFIs. Although previous studies have proposed absolute thresholds (considering an FI in the range of 19–22 to be empirically stable) [60], a relative criterion has also been used, defining trials with an SIFI ≤ 1% of the total number of enrolled patients as highly fragile [61]; however, no consensus has yet been reached regarding this criterion. Given these considerations, we adopted an operational definition in which trials with a number of WCLFU cases exceeding the absolute value of SIFI were classified as having “insufficient robustness”. This criterion provides a descriptive comparison between the observed number of patients who withdrew consent or were lost to follow-up and the number of outcome changes represented by the SIFI, rather than demonstrating that these patients would have experienced the specific outcome changes required to reverse the original statistical conclusion. Among the 50 phase III RCTs of colorectal cancer, including 9 three-arm trials, only 14 pairwise comparisons (23.73%) met this criterion, a proportion substantially lower than the 47% previously reported for RCTs in the field of acute coronary syndrome [62]. Thus, compared with the previously reported findings in acute coronary syndrome, a smaller proportion of colorectal cancer trials had an observed WCLFU count exceeding the corresponding absolute SIFI. However, when considered alongside the finding that 44.07% of pairwise comparisons had an absolute SIFI value of less than 5, these results should not be interpreted as indicating that colorectal cancer trials are generally highly robust. Rather, the findings provide a descriptive comparison of observed WCLFU and SIFI and suggest that the relationship between these two quantities may differ across disease settings. In pairwise comparisons with positive results, statistical power was found to be moderately correlated with SIFI. Trials with a power of >85% had a significantly higher median SIFI than those with a power of ≤85%, consistent with previous studies on the relationship between statistical power and traditional fragility indices [63]. One possible explanation is that high-performance trials typically have larger sample sizes and more comprehensive event data during the design phase, making them more sensitive to true effects. When such trials yield positive results, the differences between groups are often more pronounced, and the distribution of events is more stable, making them less susceptible to fluctuations caused by a small number of events; consequently, they exhibit higher SIFI values.

Among pairwise comparisons with negative results, several factors, including sample size, primary endpoint, and line of therapy, were significantly associated with SIFI. Trials with DFS as the primary endpoint had the largest absolute SIFI values, which may be related to the design characteristics of DFS as the most commonly used primary endpoint for adjuvant therapy. Compared with OS, which is influenced by subsequent treatments and typically requires a longer follow-up period for sufficient events to accrue, and PFS, which is commonly used in metastatic disease and involves relatively subjective assessments of disease progression, DFS is generally determined using more objective criteria based on imaging or pathological assessment, with relatively well-defined events. Therefore, when a trial yields a negative result, a negative DFS finding may more closely reflect the underlying treatment effect and may be less susceptible to being overturned by subsequent data, as suggested by previous literature on the characteristics of DFS [64,65,66]. With respect to line of therapy, the absolute SIFI values were significantly higher in the adjuvant therapy group than in the first-line and second-line therapy groups. This finding may be related to the lower baseline risk of recurrence and relatively lower event rates among patients receiving adjuvant therapy [67,68]. Among pairwise comparisons with negative results, there were also significant differences in the absolute values of SIFI across different colorectal cancer subtypes. Overall, pairwise comparisons for colon cancer exhibited the highest absolute negative SIFI values, suggesting that greater changes in patient outcomes would be required to alter a negative statistical conclusion. This may be related to the relatively mature adjuvant treatment regimen for colon cancer, the more standardized design of clinical trials, and the more comprehensive accumulation of events during the follow-up period [69]. In contrast, pairwise comparisons of combined colorectal cancer had the lowest absolute negative SIFI, suggesting that their statistical conclusions may be more sensitive to changes in the number of events. This phenomenon may be related to differences between colon and rectal cancers in terms of disease biology, treatment strategies, and the definition of endpoints; the combined analysis may further increase the uncertainty in interpreting the results [70]. The robustness of results from rectal cancer pairwise comparisons fell between the two, which may be related to the greater complexity of their treatment regimens, including multimodal treatment strategies involving neoadjuvant chemoradiotherapy, surgery, and adjuvant therapy [71]. These factors may influence the assessment of treatment effects and the pattern of event occurrence.

*HR* was the only trial characteristic significantly associated with SIFI in both pairwise comparisons with positive and negative results. Among pairwise comparisons with positive results, those with an *HR* < 0.75 had the highest SIFI values, suggesting that when a strong treatment effect reached statistical significance, the resulting conclusions were generally more robust. In contrast, among pairwise comparisons with negative results, the same *HR* range was associated with the lowest absolute SIFI values, suggesting that some trials showing a strong trend toward treatment benefit but failing to reach statistical significance may have conclusions that are more sensitive to changes in the number of events, sample size, or follow-up maturity. This seemingly paradoxical finding may reflect the interplay between effect size and statistical evidence: the treatment effect reflected by the *HR* determines the direction of the observed effect, whereas statistical significance depends on whether the study provides sufficient information to support that effect. Therefore, compared with relying solely on *p*-values, the *HR* point estimate may provide more clinically meaningful and methodologically informative evidence [72]. These findings also suggest that studies showing a relatively large treatment effect but insufficient statistical evidence should be interpreted cautiously, with consideration given to more mature data and further validation. This study has several strengths. First, to our knowledge, it is the first systematic evaluation of SIFI characteristics in phase III clinical trials of colorectal cancer, thereby extending previous research that has primarily relied on the conventional FI approach. Although previous studies have evaluated the FI in clinical trials of rectal cancer [73], the conventional FI is mainly based on binary event outcomes and cannot fully capture the time-to-event information and individual differences in survival represented by Kaplan–Meier curves. In contrast, by reconstructing individual-level event data and applying SIFI, our study further evaluated the impact of changes in patient outcomes on the stability of survival analysis results. This approach provides complementary information to *p*-values and *HR*s by assessing the robustness of statistical conclusions from the perspective of outcome stability. Second, unlike previous studies that have primarily focused on trials with positive results, our study included both positive and negative trials and evaluated their SIFI characteristics separately. We found that the absolute SIFI values were generally lower among trials with negative results, suggesting that some trials that failed to reach statistical significance may require only a relatively small number of changes in patient outcomes to overturn the original statistical conclusion. Compared with *p*-values, which primarily indicate the presence or absence of statistical significance, SIFI quantifies the precise “distance” to statistical significance and provides complementary information for characterizing the statistical sensitivity of trial conclusions when statistical evidence is insufficient. This approach may also inform the design and validation of subsequent studies.

This study has several limitations. First, we reconstructed individual patient-level survival data from published Kaplan–Meier curves and calculated SIFI using an unstratified log-rank framework. Because the reconstructed data were derived from published curves, some degree of reconstruction error is unavoidable, and the reconstructed *p*-values may differ from those reported in the original trials, particularly when stratified tests or Cox proportional hazards models were used. Covariate-adjusted survival analyses reported in the original trials could therefore not be fully reproduced. In addition, SIFI estimates may vary across survival analysis methods, including Cox models, restricted mean survival time, and weighted/MaxCombo approaches, particularly when the proportional hazards assumption is not met. Therefore, our SIFI estimates should be interpreted within the unstratified log-rank framework used in this study. We also used a uniform *p* < 0.05 threshold to define statistical significance. However, some phase III trials used different prespecified thresholds, including one-sided or two-sided α = 0.025, whereas others did not report a specific significance level. Thus, using a uniform threshold may have misclassified some original trial conclusions. Ideally, SIFI should be calculated using the prespecified significance threshold of each trial, but this was not feasible when the threshold was not reported. Second, SIFI primarily reflects statistical robustness and should not be interpreted as equivalent to overall trial quality or effect size. Although larger sample sizes are often associated with higher SIFI values, sample size determination is constrained by multiple factors, including study design and ethical considerations, and RCTs typically determine the minimum required sample size based on assumptions derived from prior or preliminary studies. Therefore, SIFI should not be interpreted in isolation but rather considered alongside *p*-values, 95% *CIs*, and measures of clinical effect, such as absolute risk reduction. Third, there is currently no consensus on how to define the clinical robustness of SIFI values. In this study, we used the number of WCLFU cases relative to the absolute SIFI value as an operational criterion. This approach provides a pragmatic and clinically interpretable way to relate the statistical fragility of a trial result to the actual number of patients lost to follow-up or withdrawing consent during the study, rather than relying solely on an arbitrary fixed threshold. However, this criterion has not been externally validated and should therefore be interpreted cautiously. In addition, SIFI is based on a counterfactual reassignment of patient outcomes to determine the minimum number of outcome changes required to alter the statistical conclusion. The resulting SIFI value therefore represents a measure of statistical sensitivity and does not imply that the reassigned outcomes would have actually occurred in the corresponding patients. Future studies are needed to establish more robust and generalizable criteria for interpreting SIFI values. Fourth, our analysis was restricted to phase III colorectal cancer trials published in ten selected top-tier journals to ensure a relatively consistent and well-documented evidence base. The included trials also varied in primary endpoints, treatment settings, and clinical contexts, which may contribute to heterogeneity in SIFI values. For multi-arm trials, multiple pairwise comparisons derived from the same trial were included in the analysis, and comparisons sharing a common treatment arm may be correlated because they are based on overlapping patient data. Therefore, our findings should be interpreted primarily within the context of phase III colorectal cancer trials published in these selected journals, and their generalizability may be somewhat limited. Whether the observed SIFI patterns are representative of the broader population of phase III colorectal cancer trials requires further evaluation using more comprehensive trial databases.

## 5. Conclusions

This study systematically evaluated the characteristics of SIFI in phase III clinical trials of colorectal cancer conducted between 2015 and 2025. Our findings showed that the conclusions of some trials may be sensitive to relatively small changes in patient outcomes. SIFI is not intended to replace *p*-values or measures of treatment effect; rather, by quantifying the sensitivity of statistical conclusions to changes in the underlying data, it provides complementary information on the robustness of trial results beyond conventional statistical measures. Incorporating SIFI into the reporting of future RCT results may facilitate a more comprehensive interpretation of the strength of evidence and provide additional information to inform trial design, interpretation of results, and subsequent validation studies. However, SIFI is dependent on the underlying survival analysis framework and should be interpreted as a complementary measure of statistical robustness rather than as a measure of clinical importance, treatment efficacy, bias, or overall trial quality. Further methodological research is needed to determine whether and how SIFI could contribute to sample size determination in future trials.

## Figures and Tables

**Table 1:** SIFI by Trial Characteristics Among Positive Pairwise Comparisons.

Characteristic	Frequency, *n* (%)	SIFI (M, IQR)	Statistic	*p*
**Cancer type**			3.73	0.155
Colon cancer	2 (6.9)	19.0 (15.5 to 22.5)		
Colorectal cancer	25 (86.2)	6.0 (2.0 to 11.0)		
Rectal cancer	2 (6.9)	4.0 (4.0 to 4.0)		
**Year**			2.74	0.254
2014–2017	13 (44.8)	4.0 (1.0 to 9.0)		
2018–2021	8 (27.6)	11.0 (5.5 to 12.0)		
2022–2025	8 (27.6)	4.0 (2.0 to 12.2)		
**Study type**			111.50	0.459
IIT	10 (34.5)	8.5 (4.0 to 11.2)		
IST	19 (65.5)	6.0 (1.5 to 11.5)		
**Including China**			52.50	0.385
No	23 (79.3)	4.0 (2.0 to 11.5)		
Yes	6 (20.7)	9.0 (6.5 to 11.5)		
**Blind**			87.00	0.342
No	23 (79.3)	6.0 (2.0 to 12.0)		
Yes	6 (20.7)	4.0 (1.2 to 9.0)		
**Power**			7.14	0.028
≤85%	13 (44.8)	2.0 (1.0 to 8.0)		
>85%	10 (34.5)	8.5 (2.5 to 13.5)		
NA	6 (20.7)	12.0 (7.5 to 12.8)		
**Sample size**			91.50	0.655
<500	17 (58.6)	6.0 (2.0 to 10.0)		
≥500	12 (41.4)	9.0 (1.0 to 12.5)		
**Therapeutic line**			3.99	0.262
1	8 (27.6)	2.0 (1.8 to 5.2)		
2	4 (13.8)	4.5 (1.0 to 8.8)		
Adjuvant	7 (24.1)	6.0 (3.0 to 10.5)		
Others	10 (34.5)	11.0 (6.0 to 12.8)		
**Control group**			0.38	0.828
Active control	21 (72.4)	4.0 (2.0 to 12.0)		
Observation	3 (10.3)	8.0 (5.0 to 8.5)		
Placebo or BSC alone	5 (17.2)	10.0 (6.0 to 11.0)		
**Drug classification**			2.84	0.417
Chemotherapy	8 (27.6)	4.0 (1.8 to 9.5)		
Chemotherapy + targeted therapy	8 (27.6)	5.5 (1.0 to 9.8)		
Immunotherapy	1 (3.4)	23.0 (23.0 to 23.0)		
Targeted Therapy	12 (41.4)	6.0 (2.0 to 11.2)		
**Primary endpoint**			1.69	0.640
DFS	5 (17.2)	4.0 (4.0 to 9.0)		
OS	13 (44.8)	10.0 (2.0 to 12.0)		
PFS	9 (31.0)	2.0 (2.0 to 8.0)		
Other	2 (6.9)	5.0 (3.0 to 7.0)		
**HR**			6.70	0.035
<0.75	18 (62.1)	9.0 (4.5 to 12.0)		
0.75–0.94	8 (27.6)	1.0 (1.0 to 2.5)		
≥0.95	3 (10.3)	6.0 (4.0 to 9.0)		

SIFI, survival-inferred fragility index; IIT, investigator-initiated trial; IST, industry-sponsored trial; BSC, best supportive care; OS, overall survival; DFS, disease-free survival; PFS, progression-free survival.

**Table 2:** SIFI by Trial Characteristics Among Negative Pairwise Comparisons.

Characteristic	Frequency, *n* (%)	SIFI (M, IQR)	Statistic	*p*
**Cancer type**			8.86	0.012
Colon cancer	4 (13.3)	−30.5 (−43.5 to −18.0)		
Colorectal cancer	20 (66.7)	−4.5 (−7.2 to −3.0)		
Rectal cancer	6 (20.0)	−10.5 (−11.0 to −5.5)		
**Year**			7.27	0.026
2014–2017	12 (40.0)	−6.5 (−13.5 to −4.0)		
2018–2021	8 (26.7)	−13.5 (−23.8 to −7.0)		
2022–2025	10 (33.3)	−3.5 (−5.8 to −2.2)		
**Study type**			91.00	0.574
IIT	11 (36.7)	−7.0 (−18.5 to −3.0)		
IST	19 (63.3)	−6.0 (−11.0 to −4.0)		
**Including China**			71.50	0.635
No	25 (83.3)	−6.0 (−12.0 to −3.0)		
Yes	5 (16.7)	−10.0 (−11.0 to −5.0)		
**Blind**			21.50	0.451
No	29 (96.7)	−6.0 (−12.0 to −4.0)		
Yes	1 (3.3)	−12.0 (−12.0 to −12.0)		
**Power**			3.10	0.212
≤85%	17 (56.7)	−4.0 (−11.0 to −3.0)		
>85%	9 (30.0)	−6.0 (−12.0 to −4.0)		
NA	4 (13.3)	−13.0 (−23.2 to −9.8)		
**Sample size**			163.50	0.005
<500	20 (66.7)	−4.5 (−7.2 to −3.0)		
≥500	10 (33.3)	−17.0 (−36.5 to −11.2)		
**Therapeutic line**			12.97	0.005
1	15 (50.0)	−4.0 (−8.0 to −3.0)		
2	6 (20.0)	−4.5 (−6.5 to −4.0)		
Adjuvant	7 (23.3)	−19.0 (−37.0 to −16.5)		
Others	2 (6.7)	−6.5 (−9.2 to −3.8)		
**Control group**			0.80	0.671
Active control	27 (90.0)	−6.0 (−11.5 to −3.5)		
Observation	2 (6.7)	−11.0 (−14.5 to −7.5)		
Placebo or BSC alone	1 (3.3)	−12.0 (−12.0 to −12.0)		
**Drug classification**			8.20	0.084
Chemotherapy	8 (26.7)	−15.0 (−24.8 to −9.8)		
Chemotherapy + Targeted therapy	9 (30.0)	−4.0 (−7.0 to −3.0)		
Immunotherapy	2 (6.7)	−3.0 (−3.5 to −2.5)		
Targeted Therapy	4 (13.3)	−4.5 (−7.5 to −2.5)		
Other	7 (23.3)	−8.0 (−10.5 to −4.5)		
**Primary endpoint**			15.23	<0.001
DFS	7 (23.3)	−32.0 (−40.0 to −15.5)		
OS	10 (33.3)	−6.0 (−11.0 to −4.2)		
PFS	13 (43.3)	−4.0 (−6.0 to −2.0)		
**HR**				
<0.75	4 (13.3)	−1.5 (−2.2 to −1.0)	11.47	0.003
0.75–0.94	15 (50.0)	−6.0 (−11.0 to −4.0)		
≥0.95	11 (36.7)	−12.0 (−35.0 to −6.5)		

SIFI, survival-inferred fragility index; IIT, investigator-initiated trial; IST, industry-sponsored trial; BSC, best supportive care; OS, overall survival; DFS, disease-free survival; PFS, progression-free survival.

**Table 3:** Multivariable Analysis of Factors Associated with WCLFU Exceeding the absolute value of the SIFI.

Characteristic	OR (95% *CI*)	*p*
**Power**		
≤85%	1.00 (ref)	
>85%	0.92 (0.19 to 3.93)	0.913
NA	0.33 (0.00 to 4.27)	0.441
**Study type**		
IIT	1.00 (ref)	
IST	0.69 (0.11 to 3.82)	0.672
**Including China**		
No	1.00 (ref)	
Yes	0.23 (0.02 to 1.37)	0.110
**Blind**		
No	1.00 (ref)	
Yes	3.05 (0.22 to 37.74)	0.375
**Therapeutic line**		
Others	1.00 (ref)	
1	0.72 (0.07 to 10.50)	0.785
2	1.04 (0.09 to 22.71)	0.974
Adjuvant	0.31 (0.01 to 6.88)	0.435
**Intervention drug classification**		
Chemotherapy	1.00 (ref)	
Chemotherapy + Targeted	0.30 (0.03 to 1.96)	0.214
Targeted Therapy	0.38 (0.03 to 3.59)	0.393
Immunotherapy ± Targeted	1.78 (0.10 to 31.86)	0.689
Other	1.05 (0.06 to 16.03)	0.969
**Control**		
Placebo or BSC alone	1.00 (ref)	
Active control	0.30 (0.01 to 5.45)	0.424
Observation	0.42 (0.01 to 9.71)	0.592

WCLFU, withdrawal of consent or lost to follow-up; SIFI, survival-inferred fragility index; IIT, investigator-initiated trial; IST, industry-sponsored trial; BSC, best supportive care. The analysis included 14 outcome events and 45 non-events among 59 pairwise comparisons, with an events-per-parameter ratio of 1.0.

## Data Availability

Requests to access the whole datasets regarding the manuscript should be directed to Lanwei Guo (Email: zlyyglw3609@zzu.edu.cn).

## Supplementary Materials

The following supporting information can be downloaded at: https://www.mdpi.com/article/10.3390/cancers18203394/s1. Table S1: Characteristics of the included studies; Table S2: Search strategy in PubMed; Table S3: Search strategy in Embase; Table S4: Search strategy in Cochrane; Table S5: Agreement between original and simulated results for *p* values and HR; Table S6: Comparison of Reported and Simulated Hazard Ratios and *p* Values; Table S7: SIFI by trial characteristics among positive results after excluding three-arm trials; Table S8: SIFI by trial characteristics among negative results after excluding three-arm trials; Table S9: PRISMA 2020 Checklist; Figure S1: Selection of studies outlined according to Preferred Reporting Items for Systematic Reviews and Meta-Analyses (PRISMA) guidelines; Figure S2: Correlation between SIFI and −log_10_ (*p* values) across all included colorectal cancer pairwise comparisons; Figure S3: Correlation between the absolute value of SIFI and median follow-up time for all included colorectal cancer pairwise comparisons; Figure S4: Correlation analysis between SIFI and statistical power in colorectal cancer pairwise comparisons; Figure S5: Violin plots with embedded box plots comparing SIFI values calculated using four methods; Figure S6: Correlation heatmap between SIFI and trial parameters in all included colorectal cancer pairwise comparisons; Figure S7: Correlation between SIFI and sample size after excluding three-arm trials; Figure S8: Correlation analysis between SIFI and statistical power after excluding three-arm trials.
