# Supplementary material for: Survival-Inferred Fragility Index in Phase III Colorectal Cancer Trials: A Systematic Review
Source: Cancers (Basel). 2026 Oct 20;18(20):3394. doi: 10.3390/cancers18203394 (PMC13702729; doi:10.3390/cancers18203394)
Supplement: Supplementary file 1 [file cancers-18-03394-s001.zip › cancers-4568490-supplementary.pdf]

Supplementary Online Content

Table S1 Characteristics of the included studies

Table S2 Search strategy in PubMed

Table S3 Search strategy in Embase

Table S4 Search strategy in Cochrane

Table S5 Agreement between original and simulated results for *P* values and *HR*

Table S6 Comparison of Reported and Simulated Hazard Ratios and *P* Values

Table S7. SIFI by trial characteristics among positive results after excluding three-arm trials

Table S8. SIFI by trial characteristics among negative results after excluding three-arm trials

Table S9. PRISMA 2020 Checklist

Figure S1 Selection of studies outlined according to Preferred Reporting Items for Systematic Reviews and Meta-Analyses (PRISMA) guidelines

Figure S2 Correlation between SIFI and $-\log_{10}$ (*P* values) across all included colorectal cancer pairwise comparisons

Figure S3 Correlation between the absolute value of SIFI and median follow-up time for all included colorectal cancer pairwise comparisons

Figure S4 Correlation analysis between SIFI and statistical power in colorectal cancer pairwise comparisons

Figure S5 Violin plots with embedded box plots comparing SIFI values calculated using four methods

Figure S6 Correlation heatmap between SIFI and trial parameters in all included colorectal cancer pairwise comparisons

Figure S7 Correlation between SIFI and sample size after excluding three-arm trials

Figure S8 Correlation analysis between SIFI and statistical power after excluding three-arm trials

Table S1 Characteristics of the included studies

Intervention	Drug classification	Control	Tumor type	Clinical trial/ NCT number	Study type	Year of publication	Total Sample size	Power	Median follow-up, months	Primary endpoint	*HR* (95%CI)	*P*-value[a]	WCLFU	SIFI[b]
Nivolumab + Ipilimumab	Immunotherapy	Nivolumab	Colorectal cancer	CheckMate 8HW[1]	IST	2025	707	93%	47	PFS	0.64 (0.52 to 0.79)	<0.01	0	23
Pembrolizumab	Immunotherapy	5-FU + Leucovorin + Bevacizumab/Cetuximab	Colorectal cancer	KEYNOTE-177[2]	IST	2023	307	85%	73.3	PFS	0.56 (0.26 to 1.20)	0.15	0	-2
FOLFOX + Bevacizumab	Chemotherapy + Targeted therapy	FOLFOX	Colon cancer	SAVANT(FOLFOX+bev vs FOLFOX)[3]	IST	2020	1915	NA	48	OS	1.29 (1.07 to 1.55)	0.01	0	12
XELOX + Bevacizumab	Chemotherapy + Targeted therapy	FOLFOX	Colon cancer	SAVANT(XELOX+bev vs FOLFOX)[3]	IST	2020	1907	NA	48	OS	1.15 (0.95 to 1.39)	0.14	0	-15
FOLFIRI + Bevacizumab	Chemotherapy + Targeted therapy	Observation	Colorectal cancer	PRODIGE 9[4]	IST	2018	494	90%	Intervention group:51.2; Control group:54.9	PFS	0.91 (0.76 to 1.09)	0.28	4	-4
S-1	Chemotherapy	Tegafur + Uracil	Colorectal cancer	ACTS-RC[5]	IST	2016	959	80%	60.24	RFS	0.77 (0.63 to 0.96)	0.05	0	1
FOLFIRI/FOLFOX4 + Bevacizumab	Chemotherapy + Targeted therapy	FOLFIRI/FOLFOX4	Colorectal cancer	ITACa[6]	IIT	2015	376	80%	36	PFS	0.86 (0.70 to 1.07)	0.16	8	-2

Sotorasib 960mg + Panitumumab	Targeted Therapy	Trifluridine/Tipiracil/Regorafenib	Colorectal cancer	CodeBreaK 300(Soto 960mg + Pani vs IC)[7]	IST	2025	107	70%	13.6	OS	0.70 (0.41 to 1.18)	0.18	3	-3
Sotorasib 240mg + Panitumumab	Targeted Therapy	Trifluridine/Tipiracil/Regorafenib	Colorectal cancer	CodeBreaK 300(Soto 240mg + Pani vs IC)[7]	IST	2025	107	70%	13.6	OS	0.83 (0.49 to 1.39)	0.59	3	-6
FTD-TPI + Bevacizumab	Chemotherapy + Targeted therapy	FTDTPI alone	Colorectal cancer	SUNLIGHT[8]	IIT	2023	492	90%	14.2	OS	0.61 (0.49 to 0.77)	<0.01	5	16
FOLFOX	Chemotherapy	Fluorouracil	Rectal cancer	CAO/ARO/AIO-04[9]	IIT	2015	1236	80%	50	DFS	0.79 (0.64 to 0.98)	0.04	8	4
FOLFOXIRI + Panitumumab	Chemotherapy + Targeted therapy	Leucovorin + Oxaliplatin + Panitumumab	Colorectal cancer	TRIPLETE[10]	IST	2022	435	90%	26.5	PFS	0.88 (0.70 to 1.11)	0.22	0	-4
UFT/LV 18 months	Chemotherapy	UFT/LV 6 months	Colon cancer	JFMC33-0502[11]	IIT	2015	1071	80%	33	DFS	1.00 (0.80 to 1.24)	0.98	0	-42
CAPOX/XELOX + radiotherapy (45-50.4 Gy in fractions)	Other	Capecitabine + radiotherapy	Colorectal cancer	PETACC 6[12]	IST	2020	1094	80%	68	DFS	1.02 (0.82 to 1.28)	0.86	0	-38
CAPOX/XELOX	Chemotherapy	fluorouracil/folinic acid	Colon cancer	NO16968[13]	IST	2015	1886	NA	74	DFS	0.80 (0.69 to 0.93)	<0.01	0	26
Capecitabine + Bevacizumab	Chemotherapy + Targeted therapy	Obsevation	Colorectal cancer	CAIRO3[14]	IIT	2015	558	80%	48	PFS2	0.68 (0.57 to 0.82)	<0.01	1	9

FOLFOX/CAPOX 3 mouths	Chemotherapy	6 month adjuvant FOLFOX/CAPOX	Colon cancer	HORG-IDEA[15]	IIT	2019	1115	NA	67	DFS	1.05 (0.61 to 1.55)	0.98	0	-48
Encorafenib + Binimetinib + Cetuximab	Targeted Therapy	Cetuximab + Irinotecan/Cetuximab + FOLFIRI	Colorectal cancer	BEACON(ENCO/BINI/CETUX vs control)[16]	IIT	2021	445	NA	12.8	OS	0.60 (0.47 to 0.75)	<0.01	4	13
Encorafenib + Cetuximab	Targeted Therapy	Cetuximab + Irinotecan/Cetuximab + FOLFIRI	Colorectal cancer	BEACON(ENCO/CETUX vs control)[16]	IIT	2021	441	NA	12.8	OS	0.61 (0.48 to 0.77)	<0.01	3	12
Ramucirumab + FOLFIRI	Chemotherapy + Targeted therapy	Placebo + FOLFIRI	Colorectal cancer	RAISE[17]	IST	2015	1072	85%	21.7	OS	0.844 (0.730 to 0.976)	0.04	0	1
Olaparib + Bevacizumab	Targeted therapy	Bevacizumab + 5-fluorouracil/Capecitabine	Colorectal cancer	LYNK-003(Ola + Bev vs Bev + 5-FU/Cape)[18]	IST	2024	202	NA	7.6	PFS	1.52 (1.02 to 2.27)	0.02	0	2
Olaparib	Targeted therapy	Bevacizumab + 5-fluorouracil/Capecitabine	Colorectal cancer	LYNK-003((Ola vs Bev + 5-FU/Cape))[18]	IST	2024	205	NA	7.6	PFS	2.11 (1.39 to 3.18)	<0.01	0	6
12 months Capecitabin	Chemotherapy	6 months Capecitabine	Colon cancer	JFMC37-0801[19]	IIT	2019	1304	80%	60.6	DFS	0.858 (90% CI: 0.732 to 1.004)	0.23	0	-19

Bevacizumab + Erlotinib	Targeted Therapy	Bevacizumab	Colorectal cancer	GERCOR DREAM (OPTIMOX3)[20]	IST	2015	700	80%	Intervention group:48.3; Control group:51	PFS	0.78 (0.68 to 0.96)	0.03	0	1
Nintedanib	Targeted Therapy	Placebo	Colorectal cancer	LUME-Colon 1[21]	IST	2018	768	90%	13.4	OS	1.01 (0.86 to 1.19)	0.94	0	-12
mFOLFOX6 (± bevacizumab) + SIRT	Other	mFOLFOX6 (± Bevacizumab)	Colorectal cancer	SIRFLOX[22]	IST	2016	530	80%	43.3	PFS	0.93 (0.77 to 1.12)	0.34	1	-4
Panitumumab + mFOLFOX6	Chemotherapy + Targeted therapy	Bevacizumab + mFOLFOX6	Colorectal cancer	PARADIGM[23]	IST	2023	823	80%	61.3	OS	0.84 (0.72 to 0.98)	0.04	17	1
Trifluridine/tipiracil	Chemotherapy	Placebo	Colorectal cancer	TERRA[24]	IST	2017	406	90%	13.8	OS	0.79 (0.62 to 0.99)	0.04	10	1
Capecitabine/5-FU + LV	Chemotherapy	Observation	Rectal cancer	PROCTOR-SCRIPT[25]	IIT	2015	437	90%	60	OS	0.93 (0.62 to 1.39)	0.67	0	-18
FOLFOXIRI + Bevacizumab	Chemotherapy + Targeted therapy	FOLFOX/FOLFIRI + Bevacizumab	Colorectal cancer	CAIRO5(FOLFOXIRI + Bev vs FOLFOX/FOLFIRI + Bev)[26]	IST	2023	294	80%	51.1	PFS	0.76 (0.60 to 0.98)	0.02	0	2

FOLFOX/FOLFIRI + Panitumumab	Chemotherapy + Targeted therapy	FOLFOX/FOLFIRI + Bevacizumab	Colorectal cancer	CAIRO5(FOLFOX/FOLFIRI + Pmab vs FOLFOX/FOLFIRI + Bev)[26]	IST	2023	236	80%	49.9	PFS	1.11 (0.84 to 1.48)	0.21	0	-3
50 Gy radiation therapy for 5 weeks + CAPOX/XELOX	Other	45 Gy radiation therapy for 5 weeks + Capecitabine	Rectal cancer	ACCORD12[27]	IST	2017	598	NA	60.2	DFS	0.86 (0.66 to 1.11)	0.22	0	-11
mFOLFOX	Chemotherapy	Hepatectomy Alone	Colorectal cancer	JCOG0603[28]	IIT	2021	300	80%	59.2	DFS	0.67 (0.50 to 0.92)	0.03	0	2
Oxaliplatin + Fluorouracil/Capecitabine + Bevacizumab	Chemotherapy + Targeted therapy	Fluoropyrimidine + Bevacizumab	Colorectal cancer	JCOG1018[29]	IIT	2024	251	72%	NA	PFS	0.837 (90.5% CI: 0.673 to 1.042)	0.27	0	-2
mFOLFOX6	Chemotherapy	Fluorouracil + Radiation therapy	Rectal cancer	FOWARC(mFOLFOX6 vs 5-FU + RT)[30]	IST	2024	330	81%	122.5	PFS	0.834 (0.584 to 1.190)	0.35	0	-11
mFOLFOX6 + RT	Other	Fluorouracil + Radiation therapy	Rectal cancer	FOWARC(mFOLFOX6 + RT vs 5-FU + RT)[30]	IST	2024	330	81%	122.5	PFS	0.862 (0.606 to 1.226)	0.31	0	-10

short-course radiotherapy (5×5 Gy) + FOLFOX	Other	50.4 Gy + FOLFOX	Rectal cancer	NCT00833131 [31]	IIT	2016	541	80%	35	OS	0.73 (0.53 to 1.01)	0.06	2	-1
FOLFOXIRI + Capecitabine	Chemotherapy	Capecitabine + FOLFOX	Rectal cancer	UNICANCER -PRODIGE 23[32]	IIT	2021	461	90%	46.5	DFS	0.69 (0.49 to 0.97)	0.03	8	4
FOLFOXIRI + Bevacizumab	Chemotherapy + Targeted therapy	mFOLFOX6	Colorectal cancer	TRIBE2[33]	IIT	2017	679	80%	35.9	DFS	0.74 (0.63 to 0.88)	<0.01	0	9
FOLFIRI	Chemotherapy	5-FU/LV	Rectal cancer	French R98[34]	IIT	2015	357	80%	156	PFS	0.80	0.154	13	-4
Fruquintinib	Targeted therapy	Placebo	Colorectal cancer	FRESCO-2[35]	IST	2023	691	90%	Intervention group:11.3; Control group:11.2	PFS	0.66 (0.55 to 0.80)	<0.01	24	11
Atezolizumab + Cobimetinib	Immunotherapy + Targeted therapy	Regorafenib	Colorectal cancer	IMblaze370(Atezo + Cobi vs Rego)[36]	IST	2019	273	80%	7,3	OS	1.00 (0.73 to 1.38)	0.85	14	-8
Atezolizumab	Immunotherapy	Regorafenib	Colorectal cancer	IMblaze370(Atezo vs Rego)[36]	IST	2019	180	80%	7,4	OS	1.19 (0.83 to 1.71)	0.34	7	-4
Sotorasib 240 mg + Panitumumab	Targeted Therapy	Trifluridine–tipiracil/Regorafenib	Colorectal cancer	KRAS G12C(Soto 240 + Pmab vs TFTD / Rego)[37]	IST	2023	107	90%	7.8	PFS	0.59 (0.37 to 0.95)	0.08	2	-1

Sotorasib 960 mg + Panitumumab	Targeted Therapy	Trifluridine–tipiracil/Regorafenib	Colorectal cancer	KRAS G12C(Soto 960 + Pmab vs TFTD / Rego)[37]	IST	2023	107	90%	7.8	PFS	0.48 (0.30 to 0.78)	<0.01	0	2
FLOX + Cetuximab	Chemotherapy + Targeted therapy	FLOX	Colorectal cancer	NORDIC-VII[38]	IST	2017	372	NA	100	OS	0.92 (0.74 to 1.14)	0.58	0	-6
FOLFOX7–FOLFIRI	Chemotherapy	FOLFOX4	Colorectal cancer	MIROX[39]	IIT	2014	284	80%	67	DFS	0.94 (0.70 to 1.26)	0.77	0	-12
Bevacizumab 10 mg/kg + FOLFIRI	Chemotherapy + Targeted therapy	Bevacizumab 5 mg/kg + FOLFIRI	Colorectal cancer	EAGLE[40]	IIT	2015	369	90%	13.1	PFS	0.95 (0.75 to 1.21)	0.91	2	-7
Lenvatinib + Pembrolizumab	Immunotherapy + Targeted therapy	Regorafenib/Trifluridine/Tipiracil	Colorectal cancer	LEAP-017[41]	IST	2024	480	90%	18.6	OS	0.83 (0.68 to 1.02)	0.2	0	-5
Capecitabine + Bevacizumab	Chemotherapy + Targeted therapy	Capecitabine	Colorectal cancer	QUASAR 2[42]	IST	2016	1952	90%	59.4	DFS	1.06 (0.89 to 1.25)	0.3	2	-32
Panitumumab + BSC	Targeted Therapy	BSC	Colorectal cancer	NCT01412957 [43]	IST	2016	270	90%	Intervention group:9.43; Control group:5.87	OS	0.70 (0.53 to 0.93)	0.02	4	2
Encorafenib + Binimetinib + Cetuximab	Targeted Therapy	Cetuximab + Irinotecan/Cetuximab+FOLFIRI	Colorectal cancer	BEACON(ENCO + BINI + CET vs CET + IRI/CET + FOLFIRI)[44]	IST	2019	445	90%	7.8	OS	0.52 (0.39 to 0.70)	<0.01	4	12

Encorafenib + Cetuximab	Targeted Therapy	Cetuximab + Irinotecan/Cetuximab+FOLFIRI	Colorectal cancer	BEACON(ENCO + CET vs CET + IRI/CET + FOLFIRI))[44]	IST	2019	441	90%	7.8	OS	0.60 (0.45 to 0.79)	<0.01	3	6
S-1	Chemotherapy	Capecitabine	Colorectal cancer	SALTO[45]	IIT	2017	161	90%	20.2	PFS	0.99 (0.71 to 1.37)	0.94	0	-6
Regorafenib	Targeted Therapy	Placebo	Colorectal cancer	CONCUR[46]	IST	2015	204	80%	7.4	OS	0.55 (0.40 to 0.77)	<0.01	5	6
Fruquintinib + BSC	Targeted Therapy	Placebo + BSC	Colorectal cancer	FRESCO[47]	IST	2018	416	80%	Intervention group:13.3; Control group:13.2	OS	0.65 (0.51 to 0.83)	<0.01	12	10
Capecitabine	Chemotherapy	Observation	Colorectal cancer	NCT02027363 [48]	IIT	2016	274	80%	29	PFS	0.54 (0.42 to 0.70)	<0.01	0	8
FOLFIRI/mFOLFOX-6 + Bevacizumab	Chemotherapy + Targeted therapy	mFOLFOX6/FOLFIRI	Colorectal cancer	BEBYP[49]	IIT	2014	184	80%	45.3	PFS	0.70 (0.52 to 0.95)	0.03	0	1
TAS-102	Chemotherapy	Placebo	Colorectal cancer	TAS-102[50]	IST	2015	800	90%	11.8	OS	0.68 (0.58 to 0.81)	<0.01	15	14

WCLFU, withdrawal of consent or lost to follow-up; IIT, Investigator-Initiated Trial; IST, Industry-sponsored trial; OS, Overall Survival; PFS, Progression-Free Survival; RFS, Relapse-Free Survival; DFS, Disease-Free Survival; BSC, Best Supportive Care; SIFI, Survival-inferred fragility index

[a] Calculated using 2-sided unstratified log-rank test.

[b] Survival-inferred fragility index associated with the calculated *P* value (α = 0.05)

Table S2 Search strategy in PubMed

Number	Concept	Strategy
1	Disease (Cancer-related terms)	("cancer*"[MeSH Terms] OR "cancer*"[Title/Abstract] OR "neoplas*"[Title/Abstract] OR "tumo*"[Title/Abstract] OR "carcinoma*"[Title/Abstract] OR "hodgkin*"[Title/Abstract] OR "nonhodgkin*"[Title/Abstract] OR "adenocarcinoma*"[Title/Abstract] OR ("leuk"[All Fields] AND "emia*"[Title/Abstract]) OR "metasta*"[Title/Abstract] OR "malignan*"[Title/Abstract] OR "lymphoma*"[Title/Abstract] OR "sarcoma*"[Title/Abstract] OR "melanoma*"[Title/Abstract] OR "myeloma*"[Title/Abstract] OR "oncolog*"[Title/Abstract] OR "mass*"[Title/Abstract])
2	Study design (Randomized controlled trials)	("randomized controlled trial"[Title/Abstract] OR "controlled clinical trial"[Title/Abstract] OR "randomly"[Title/Abstract] OR "randomization"[Title/Abstract] OR "random*"[Title/Abstract] OR "random allocation"[Title/Abstract] OR "placebo"[Title/Abstract] OR "placebo*"[Title/Abstract] OR "trial"[Title/Abstract] OR "groups"[Title/Abstract] OR "control*"[Title/Abstract] OR "double-blind"[Title/Abstract] OR "single-blind"[Title/Abstract] OR "clinical trial"[Title/Abstract] OR "comparative"[Title/Abstract] OR "evaluation"[Title/Abstract] OR "follow-up"[Title/Abstract] OR "prospectiv*"[Title/Abstract])
3	Outcomes (Survival endpoints)	("overall survival"[Title/Abstract] OR "progression free survival"[Title/Abstract] OR "PFS"[Title/Abstract] OR "OS"[Title/Abstract] OR "Kaplan-Meier"[Title/Abstract] OR "K-M"[Title/Abstract] OR "KM"[Title/Abstract] OR "Cox"[Title/Abstract] OR "hazard ratio"[Title/Abstract] OR "hazard*"[Title/Abstract])
4	Trial phase	(("iii"[Title/Abstract] OR "3"[Title/Abstract]) AND "phase"[Title/Abstract])
5	Journal restriction	("The New England Journal of Medicine"[Journal] OR "The Lancet"[Journal] OR "JAMA"[Journal] OR "BMJ"[Journal] OR "Journal of Clinical Oncology"[Journal] OR "The Lancet Oncology"[Journal] OR "Annals of Oncology"[Journal] OR "British Journal of Cancer"[Journal] OR "European Journal of Cancer"[Journal] OR "JAMA Oncology"[Journal])
6	Time restriction	(y_10[Filter])

Table S3 Search strategy in Embase

Number	Concept	Strategy
1	Disease (Malignant neoplasms)	('malignant neoplasm'/exp OR 'cancer' OR 'cancers' OR 'malignant neoplasia' OR 'malignant neoplasm' OR 'malignant neoplastic disease' OR 'malignant tumor' OR 'malignant tumour' OR 'neoplasia, malignant' OR 'neoplasmic malignancy' OR 'neoplastic malignancy' OR 'oncologic malignancy' OR 'oncological malignancy' OR 'tumor, malignant' OR 'tumoral malignancy' OR 'tumorous malignancy' OR 'tumour, malignant')
2	Outcomes (Survival analysis)	('hazard ratio'/exp OR 'hazard ratio' OR 'proportional hazards model'/exp OR 'cox model' OR 'cox regression' OR 'cox survival model' OR 'proportional hazards model' OR 'proportional hazards regression' OR 'kaplan meier method'/exp OR 'kaplan meier analysis' OR 'kaplan meier curve' OR 'kaplan meier estimator' OR 'kaplan-meier analysis' OR 'kaplan-meier curve' OR 'kaplan-meier estimator')
3	Study design + Phase	('randomized controlled trial'/exp OR 'randomized controlled trial' OR 'randomised controlled trial' OR 'phase 3 clinical trial'/exp OR 'phase 3 clinical trial' OR 'phase iii clinical trial')
4	Time restriction	[2015–2025]/py
5	Journal restriction	('annals of oncology'/jt OR 'british journal of cancer'/jt OR 'british medical journal'/jt OR 'european journal of cancer'/jt OR 'jama'/jt OR 'jama oncology'/jt OR 'journal of clinical oncology'/jt OR 'lancet'/jt OR 'lancet oncology'/jt OR 'new england journal of medicine'/jt)

Table S4 Search strategy in Cochrane

Number	Concept	Strategy
1	Disease (MeSH)	MeSH descriptor: [Neoplasms] explode all trees
2	Disease (Free-text)	(cancer* OR neoplas* OR tumor* OR tumour* OR carcinoma* OR malignan* OR lymphoma* OR sarcoma* OR melanoma* OR myeloma* OR oncolog*):ti,ab,kw
3	Study design (RCT)	(random* OR placebo* OR trial OR control*):ti,ab,kw
4	Outcomes (Survival)	('overall survival' OR 'progression-free survival' OR PFS OR OS OR 'hazard ratio' OR hazard* OR cox OR 'Kaplan-Meier'):ti,ab,kw
5	Phase (III/3)	(III OR 3):ti,ab,kw
6	Phase (keyword)	(phase):ti,ab,kw
7	Phase combination	#5 AND #6
8	Disease combination	#1 OR #2
9	Final search	#8 AND #3 AND #4 AND #7 with Publication Year from 2015 to 2025, in Trials

Table S5 Agreement between original and simulated results for *P* values and *HR*

Measure	*P* values	*HR* (log scale)
ICC (95% CI)	0.796 (95%CI: 0.675 to 0.875)	0.856 (95%CI: 0.764 to 0.912)
P value (ICC)	<0.001	<0.001
Mean difference	0.019	-0.042
Lower limit of agreement	-0.387	-0.323
Upper limit of agreement	0.425	0.240

ICC, Intraclass Correlation Coefficient

Table S6 Comparison of Reported and Simulated Hazard Ratios and *P* Values

Clinical trial/NCT number	Reported *HR*	Simulated *HR*	Reported *P*	Simulated *P*
CheckMate 8HW[1]	0.64	0.65	NA	<0.01
KEYNOTE-177[2]	0.56	0.57	NA	0.15
SAVANT(FOLFOX+bev vs FOLFOX)[3]	1.29	1.27	0.008	0.01
SAVANT(XELOX+bev vs FOLFOX)[3]	1.15	1.16	0.147	0.14
PRODIGE 9[4]	0.91	0.9	0.316	0.28
ACTS-RC[5]	0.77	1.23	0.0165	0.05
ITACa[6]	0.86	0.86	0.182	0.16
CodeBreaK 300(Soto 960mg + Pani vs IC)[7]	0.70	0.69	0.2	0.18
CodeBreaK 300(Soto 240mg + Pani vs IC)[7]	0.83	0.87	0.5	0.59
SUNLIGHT[8]	0.61	0.62	<0.001	<0.01
CAO/ARO/AIO-04[9]	0.79	0.8	0.03	0.04
TRIPLETE[10]	0.88	0.87	0.277	0.22
JFMC33-0502[11]	1.00	1	0.98	1
PETACC 6[12]	1.02	1.02	0.835	0.86
NO16968[13]	0.80	0.8	0.004	<0.01
CAIRO3[14]	0.68	0.69	<0.0001	<0.01
HORG-IDEA[15]	1.05	1	0.647	0.98
BEACON(ENCO/BINI/CETUX vs control)[16]	0.60	0.59	NA	<0.01
BEACON(ENCO/CETUX vs control)[16]	0.61	0.6	NA	<0.01
RAISE[17]	0.844	0.86	0.0219	0.04
LYNK-003(Ola + Bev vs Bev + 5-FU/Cape)[18]	1.52	1.61	0.982	0.02
LYNK-003(Ola vs Bev + 5-FU/Cape)[18]	2.11	2.12	0.999	<0.01
JFMC37-0801[19]	0.858	0.89	0.0549	0.23
GERCOR DREAM (OPTIMOX3)[20]	0.78	1.27	0.019	0.03
LUME-Colon 1[21]	1.01	0.99	0.8659	0.94
SIRFLOX[22]	0.93	0.92	0.43	0.34
PARADIGM[23]	0.84	0.85	0.03	0.04
TERRA[24]	0.79	0.78	0.035	0.04
PROCTOR-SCRIPT[25]	0.93	0.92	0.73	0.67
CAIRO5(FOLFOXIRI + Bev vs FOLFOX/FOLFIRI + Bev)[26]	0.76	1.35	0.032	0.02

CAIRO5(FOLFOX/FOLFIRI + Pmab vs FOLFOX/FOLFIRI + Bev)[26]	1.11	0.84	0.46	0.21
ACCORD12[27]	0.86	0.85	0.25	0.22
JCOG0603[28]	0.67	0.7	0.006	0.03
JCOG1018[29]	0.837	0.87	0.086	0.27
FOWARC(mFOLFOX6 vs 5-FU + RT)[30]	0.834	0.85	0.317	0.35
FOWARC(mFOLFOX6 + RT vs 5-FU + RT)[30]	0.862	0.83	0.408	0.31
NCT00833131[31]	0.73	1.38	0.046	0.06
UNICANCER-PRODIGE 23[32]	0.69	0.68	0.034	0.03
TRIBE2[33]	0.74	0.75	<0.001	<0.01
French R98[34]	0.8	0.81	0.154	0.17
FRESCO-2[35]	0.66	0.68	<0.0001	<0.01
IMblaze370(Atezo + Cobi vs Rego)[36]	1	1.03	0.99	0.85
IMblaze370(Atezo vs Rego)[36]	1.19	1.19	0.34	0.34
KRAS G12C(Soto 240 + Pmab vs TFTD / Rego)[37]	0.59	0.65	0.036	0.08
KRAS G12C(Soto 960 + Pmab vs TFTD / Rego)[37]	0.48	0.51	0.005	< 0.01
NORDIC-VII[38]	0.92	0.94	0.43	0.58
MIROX[39]	0.94	0.96	0.679	0.77
EAGLE[40]	0.95	1.01	0.676	0.91
LEAP-017[41]	0.83	0.87	0.0379	0.2
QUASAR 2[42]	1.06	1.1	0.54	0.3
NCT01412957[43]	0.70	0.74	0.0135	0.02
BEACON(ENCO + BINI + CET vs CET + IRI/CET + FOLFIRI)[44]	0.52	0.54	<0.001	<0.01
BEACON(ENCO + CET vs CET + IRI/CET + FOLFIRI))[44]	0.60	0.63	<0.001	<0.01
SALTO[45]	0.99	0.99	0.93	0.94
CONCUR[46]	0.55	0.54	0.00016	<0.01
FRESCO[47]	0.65	0.61	<0.001	<0.01
NCT02027363[48]	0.54	0.54	<0.001	<0.01
BEBYP[49]	0.7	0.71	0.01	0.03
TAS-102[50]	0.68	0.68	<0.001	<0.01

HR, hazard ratios; NA, not available

Table S7 SIFI by trial characteristics among positive results after excluding three-arm trials

Characteristic	Frequency, *n* (%)	SIFI (M, IQR)	Statistic	*P*
**Cancer type**			2.81	0.245
colon cancer	1 (5.0)	26.0 (26.0 to 26.0)		
colorectal cancer	17 (85.0)	6.0 (1.0 to 10.0)		
rectal cancer	2 (10.0)	4.0 (4.0 to 4.0)		
**Year**			2.06	0.357
2014-2017	13 (65.0)	4.0 (1.0 to 9.0)		
2018-2021	3 (15.0)	4.0 (3.0 to 7.0)		
2022-2025	4 (20.0)	13.5 (8.5 to 17.8)		
**Study type**			45.00	0.845
IIT	8 (40.0)	6.0 (3.5 to 9.0)		
IST	12 (60.0)	4.0 (1.0 to 11.8)		
**Including China**			45.50	0.507
No	15 (75.0)	4.0 (1.0 to 10.0)		
Yes	5 (25.0)	8.0 (6.0 to 10.0)		
**Blind**			48.50	0.616
No	14 (70.0)	6.0 (1.2 to 12.8)		
Yes	6 (30.0)	4.0 (1.2 to 9.0)		
**Power**			5.59	0.061
≤85%	12 (60.0)	3.0 (1.0 to 8.2)		
＞85%	7 (35.0)	11.0 (3.0 to 15.0)		
NA	1 (5.0)	26.0 (26.0 to 26.0)		
**Sample size**			56.50	0.616
<500	9 (45.0)	4.0 (2.0 to 8.0)		
≥500	11 (55.0)	9.0 (1.0 to 12.5)		
**Therapeutic line**			1.74	0.629
1	7 (35.0)	2.0 (1.5 to 6.5)		
2	4 (20.0)	4.5 (1.0 to 8.8)		
Adjuvant	4 (20.0)	6.5 (3.2 to 13.2)		
Others	5 (25.0)	10.0 (6.0 to 14.0)		
**Control Group**			0.93	0.627
Active control	12 (60.0)	3.0 (1.0 to 10.8)		
Observation	3 (15.0)	8.0 (5.0 to 8.5)		
Placebo or BSC alone	5 (25.0)	10.0 (6.0 to 11.0)		
**Drug classification**			2.41	0.492
Chemotherapy	8 (40.0)	4.0 (1.8 to 9.5)		
Chemotherapy+Targeted therapy	6 (30.0)	5.0 (1.0 to 9.0)		
Immunotherapy	1 (5.0)	23.0 (23.0 to 23.0)		
Targeted Therapy	5 (25.0)	6.0 (2.0 to 10.0)		
**Primary endpoint**			0.67	0.880
DFS	5 (25.0)	4.0 (4.0 to 9.0)		
OS	8 (40.0)	4.0 (1.0 to 11.0)		

PFS	5 (25.0)	8.0 (1.0 to 11.0)		
Other	2 (10.0)	5.0 (3.0 to 7.0)		
**HR**			72.00	0.037
<0.75	13 (65.0)	9.0 (4.0 to 11.0)		
0.75-0.94	7 (35.0)	1.0 (1.0 to 2.5)		

SIFI, survival-inferred fragility index; IIT, investigator-initiated trial; IST, industry-sponsored trial; BSC, best supportive care; OS, overall survival; DFS, disease-free survival; PFS, progression-free survival.

Table S8 SIFI by trial characteristics in negative results after excluding three-arm trials

Characteristic	Frequency, *n* (%)	SIFI (M, IQR)	Statistic	*P*
**Cancer type**			6.41	0.041
colon cancer	3 (14.3)	-42.0 (-45.0 to -30.5)		
colorectal cancer	14 (66.7)	-5.5 (-10.8 to -4.0)		
rectal cancer	4 (19.0)	-7.5 (-12.8 to -3.2)		
**Year**			6.10	0.047
2014-2017	12 (57.1)	-6.5 (-13.5 to -4.0)		
2018-2021	5 (23.8)	-19.0 (-38.0 to -12.0)		
2022-2025	4 (19.0)	-3.0 (-4.2 to -2.0)		
**Study type**			58.50	0.832
IIT	11 (52.4)	-7.0 (-18.5 to -3.0)		
IST	10 (47.6)	-5.5 (-11.8 to -4.0)		
**Including China**			12.50	0.470
No	19 (90.5)	-7.0 (-18.5 to -4.0)		
Yes	2 (9.5)	-4.5 (-4.8 to -4.2)		
**Blind**			13.50	0.618
No	20 (95.2)	-6.0 (-18.2 to -4.0)		
Yes	1 (4.8)	-12.0 (-12.0 to -12.0)		
**Power**			1.95	0.377
≤85%	10 (47.6)	-4.0 (-17.2 to -2.0)		
＞85%	8 (38.1)	-6.5 (-13.5 to -4.8)		
NA	3 (14.3)	-11.0 (-29.5 to -8.5)		
**Sample size**			85.00	0.029
<500	12 (57.1)	-4.5 (-6.2 to -3.5)		
≥500	9 (42.9)	-19.0 (-38.0 to -11.0)		
**Therapeutic line**			10.46	0.015
1	10 (47.6)	-4.0 (-6.0 to -2.5)		
2	4 (19.0)	-4.5 (-5.5 to -3.2)		
Adjuvant	6 (28.6)	-25.5 (-39.5 to -18.2)		
Others	1 (4.8)	-12.0 (-12.0 to -12.0)		
**Control Group**			0.35	0.840
Active control	18 (85.7)	-6.0 (-17.2 to -4.0)		
Observation	2 (9.5)	-11.0 (-14.5 to -7.5)		
Placebo or BSC alone	1 (4.8)	-12.0 (-12.0 to -12.0)		
**Drug classification**			6.42	0.170
Chemotherapy	7 (33.3)	-18.0 (-30.5 to -9.0)		
Chemotherapy+Targeted therapy	7 (33.3)	-4.0 (-6.5 to -3.0)		
Immunotherapy	1 (4.8)	-2.0 (-2.0 to -2.0)		
Targeted Therapy	1 (4.8)	-12.0 (-12.0 to -12.0)		
Other	5 (23.8)	-5.0 (-11.0 to -4.0)		
**Primary endpoint**			12.87	0.002
DFS	7 (33.3)	-32.0 (-40.0 to -15.5)		
OS	5 (23.8)	-6.0 (-12.0 to -5.0)		

PFS	9 (42.9)	-4.0 (-4.0 to -2.0)		
**HR**			10.54	0.005
<0.75	2 (9.5)	-1.5 (-1.8 to -1.2)		
0.75-0.94	12 (57.1)	-4.5 (-11.2 to -4.0)		
≥0.95	7 (33.3)	-32.0 (-40.0 to -9.5)		

SIFI, survival-inferred fragility index; IIT, investigator-initiated trial; IST, industry-sponsored trial; BSC, best supportive care; OS, overall survival; DFS, disease-free survival; PFS, progression-free survival.

TableS9 PRISMA 2020 Checklist

Section and Topic	Item #	Checklist item	Location where item is reported
**TITLE**			
Title	1	Identify the report as a systematic review.	Page1
**ABSTRACT**			
Abstract	2	See the PRISMA 2020 for Abstracts checklist.	Page2
**INTRODUCTION**			
Rationale	3	Describe the rationale for the review in the context of existing knowledge.	Page2-3
Objectives	4	Provide an explicit statement of the objective(s) or question(s) the review addresses.	Page3
**METHODS**			
Eligibility criteria	5	Specify the inclusion and exclusion criteria for the review and how studies were grouped for the syntheses.	Page3-4
Information sources	6	Specify all databases, registers, websites, organisations, reference lists and other sources searched or consulted to identify studies. Specify the date when each source was last searched or consulted.	Page3
Search strategy	7	Present the full search strategies for all databases, registers and websites, including any filters and limits used.	Page3
Selection process	8	Specify the methods used to decide whether a study met the inclusion criteria of the review, including how many reviewers screened each record and each report retrieved, whether they worked independently, and if applicable, details of automation tools used in the process.	Page3-4
Data collection process	9	Specify the methods used to collect data from reports, including how many reviewers collected data from each report, whether they worked independently, any processes for obtaining or confirming data from study investigators, and if applicable, details of automation tools used in the process.	Page3-4
Data items	10a	List and define all outcomes for which data were sought. Specify whether all results that were compatible with each outcome domain in each study	Page3-4

Section and Topic	Item #	Checklist item	Location where item is reported
		were sought (e.g. for all measures, time points, analyses), and if not, the methods used to decide which results to collect.	
	10b	List and define all other variables for which data were sought (e.g. participant and intervention characteristics, funding sources). Describe any assumptions made about any missing or unclear information.	Page3-4
Study risk of bias assessment	11	Specify the methods used to assess risk of bias in the included studies, including details of the tool(s) used, how many reviewers assessed each study and whether they worked independently, and if applicable, details of automation tools used in the process.	Page3-4
Effect measures	12	Specify for each outcome the effect measure(s) (e.g. risk ratio, mean difference) used in the synthesis or presentation of results.	Page3-4
Synthesis methods	13a	Describe the processes used to decide which studies were eligible for each synthesis (e.g. tabulating the study intervention characteristics and comparing against the planned groups for each synthesis (item #5)).	Page4-5
	13b	Describe any methods required to prepare the data for presentation or synthesis, such as handling of missing summary statistics, or data conversions.	Pag4-5
	13c	Describe any methods used to tabulate or visually display results of individual studies and syntheses.	Page4-5
	13d	Describe any methods used to synthesize results and provide a rationale for the choice(s). If meta-analysis was performed, describe the model(s), method(s) to identify the presence and extent of statistical heterogeneity, and software package(s) used.	Page4-5
	13e	Describe any methods used to explore possible causes of heterogeneity among study results (e.g. subgroup analysis, meta-regression).	Page4-5
	13f	Describe any sensitivity analyses conducted to assess robustness of the synthesized results.	Page4-5
Reporting bias	14	Describe any methods used to assess risk of bias due to missing results in a synthesis (arising from reporting	N/A

Section and Topic	Item #	Checklist item	Location where item is reported
assessment		biases).	
Certainty assessment	15	Describe any methods used to assess certainty (or confidence) in the body of evidence for an outcome.	N/A
**RESULTS**			
Study selection	16a	Describe the results of the search and selection process, from the number of records identified in the search to the number of studies included in the review, ideally using a flow diagram.	Page9
	16b	Cite studies that might appear to meet the inclusion criteria, but which were excluded, and explain why they were excluded.	Page5
Study characteristics	17	Cite each included study and present its characteristics.	Page5
Risk of bias in studies	18	Present assessments of risk of bias for each included study.	N/A
Results of individual studies	19	For all outcomes, present, for each study: (a) summary statistics for each group (where appropriate) and (b) an effect estimate and its precision (e.g. confidence/credible interval), ideally using structured tables or plots.	Page5-10
Results of syntheses	20a	For each synthesis, briefly summarise the characteristics and risk of bias among contributing studies.	Page5-10
	20b	Present results of all statistical syntheses conducted. If meta-analysis was done, present for each the summary estimate and its precision (e.g. confidence/credible interval) and measures of statistical heterogeneity. If comparing groups, describe the direction of the effect.	Page5-10
	20c	Present results of all investigations of possible causes of heterogeneity among study results.	Page5-10
	20d	Present results of all sensitivity analyses conducted to assess the robustness of the synthesized results.	Page10-11
Reporting biases	21	Present assessments of risk of bias due to missing results (arising from reporting biases) for each synthesis assessed.	Page10-11
Certainty of	22	Present assessments of certainty (or confidence) in the	Page10-

Section and Topic	Item #	Checklist item	Location where item is reported
evidence		body of evidence for each outcome assessed.	11
**DISCUSSION**			
Discussion	23a	Provide a general interpretation of the results in the context of other evidence.	Page11
	23b	Discuss any limitations of the evidence included in the review.	Page13-14
	23c	Discuss any limitations of the review processes used.	Page13-14
	23d	Discuss implications of the results for practice, policy, and future research.	Page13
**OTHER INFORMATION**			
Registration and protocol	24a	Provide registration information for the review, including register name and registration number, or state that the review was not registered.	Page3
	24b	Indicate where the review protocol can be accessed, or state that a protocol was not prepared.	N/A
	24c	Describe and explain any amendments to information provided at registration or in the protocol.	N/A
Support	25	Describe sources of financial or non-financial support for the review, and the role of the funders or sponsors in the review.	Page14
Competing interests	26	Declare any competing interests of review authors.	Page15
Availability of data, code and other materials	27	Report which of the following are publicly available and where they can be found: template data collection forms; data extracted from included studies; data used for all analyses; analytic code; any other materials used in the review.	Page15

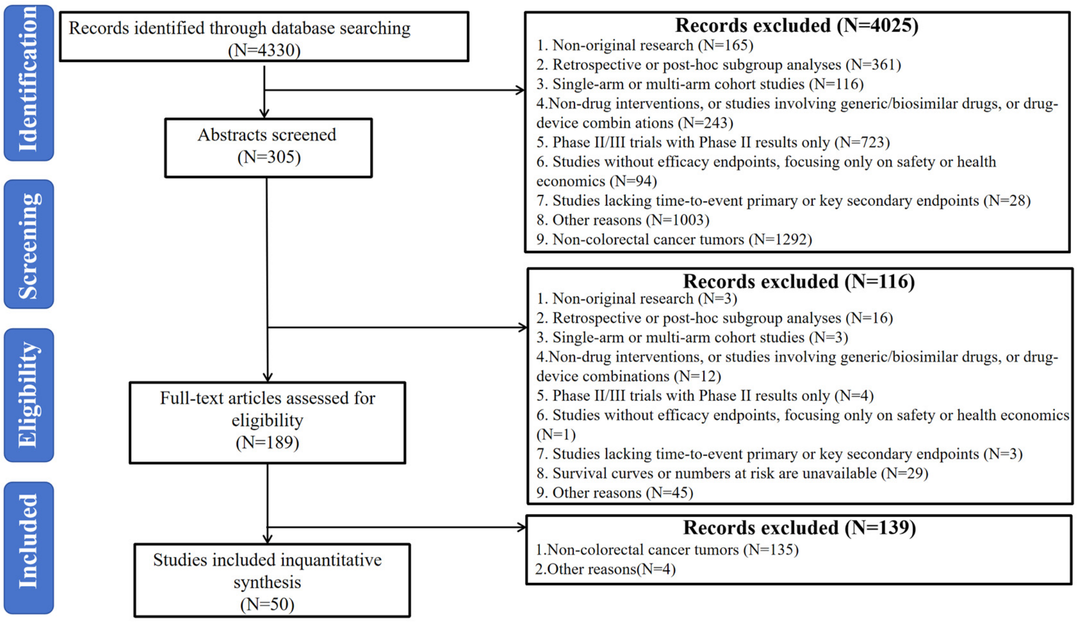

Figure S1 Selection of studies outlined according to Preferred Reporting Items for Systematic Reviews and Meta-Analyses (PRISMA) guidelines

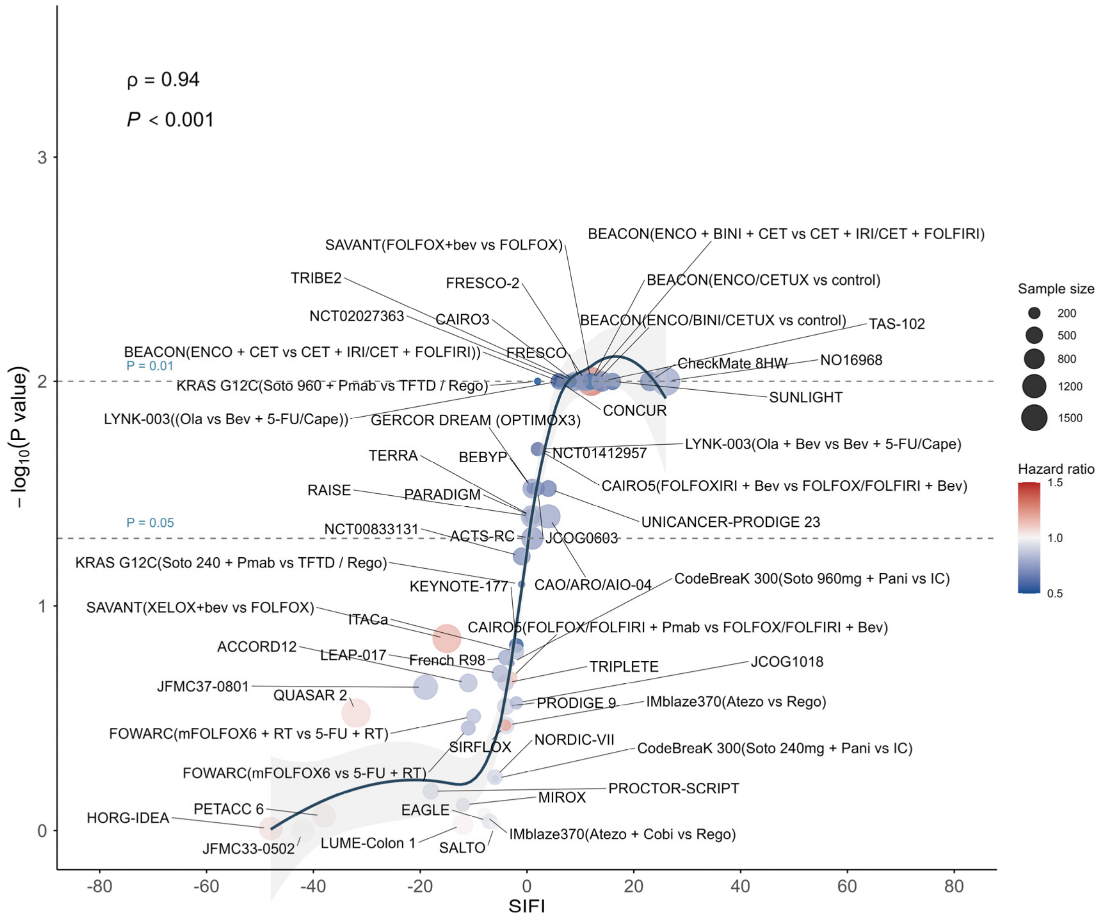

Figure S2 Correlation between SIFI and -log$_{10}$ (P values) across all included colorectal cancer pairwise comparisons.

Each point represents an individual trial, with point size proportional to sample size and color indicating the hazard ratio. The solid blue line represents the LOESS fit, and the horizontal dashed line indicates the conventional significance threshold. SIFI, survival-inferred fragility index; LOESS, locally estimated scatterplot smoothing.

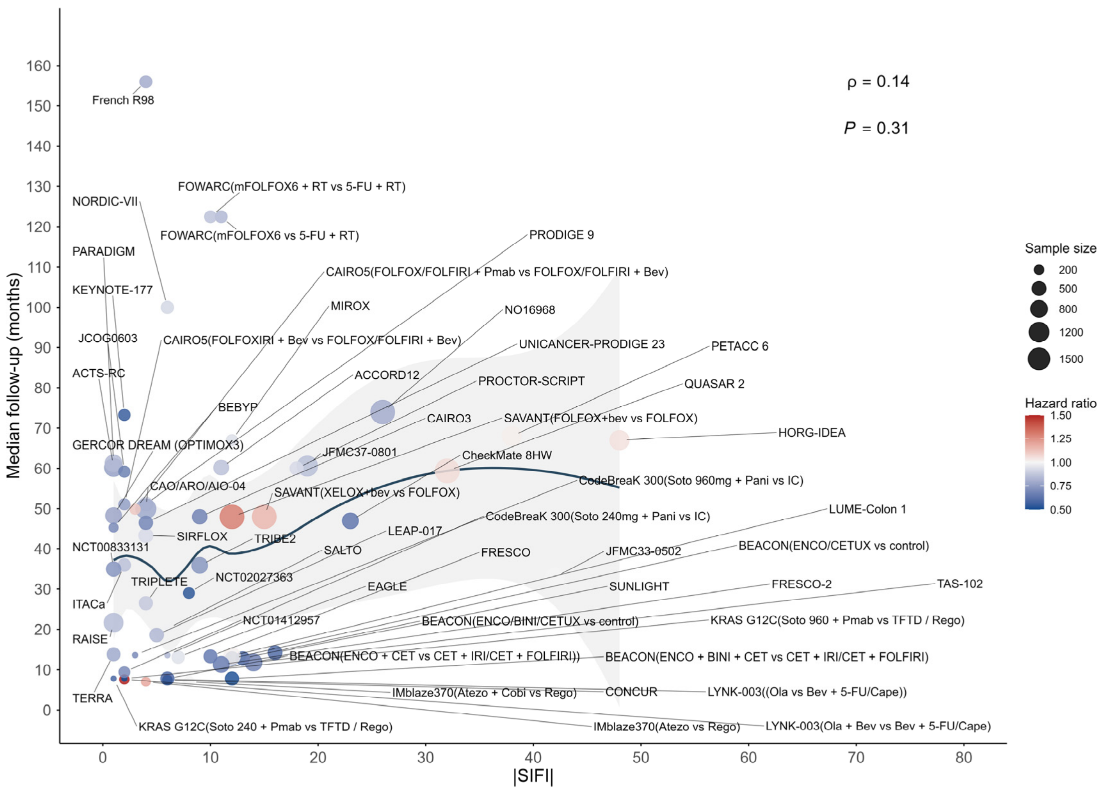

Figure S3 Correlation between the absolute value of SIFI and median follow-up time for all included colorectal cancer pairwise comparisons.

Each point represents an individual trial, with point size proportional to sample size and color indicating the hazard ratio. The solid blue line represents the LOESS fit, and the horizontal dashed line indicates the conventional significance threshold. SIFI, survival-inferred fragility index; LOESS, locally estimated scatterplot smoothing.

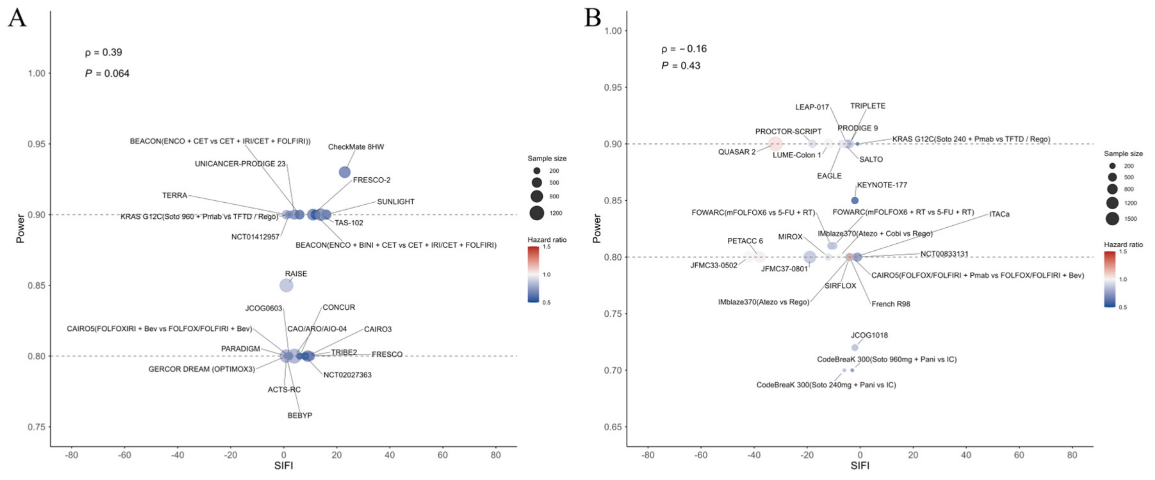

Figure S4 Correlation analysis between SIFI and statistical power in colorectal cancer pairwise comparisons. (A) Positive trials, (B) Negative trials

SIFI, survival-inferred fragility index

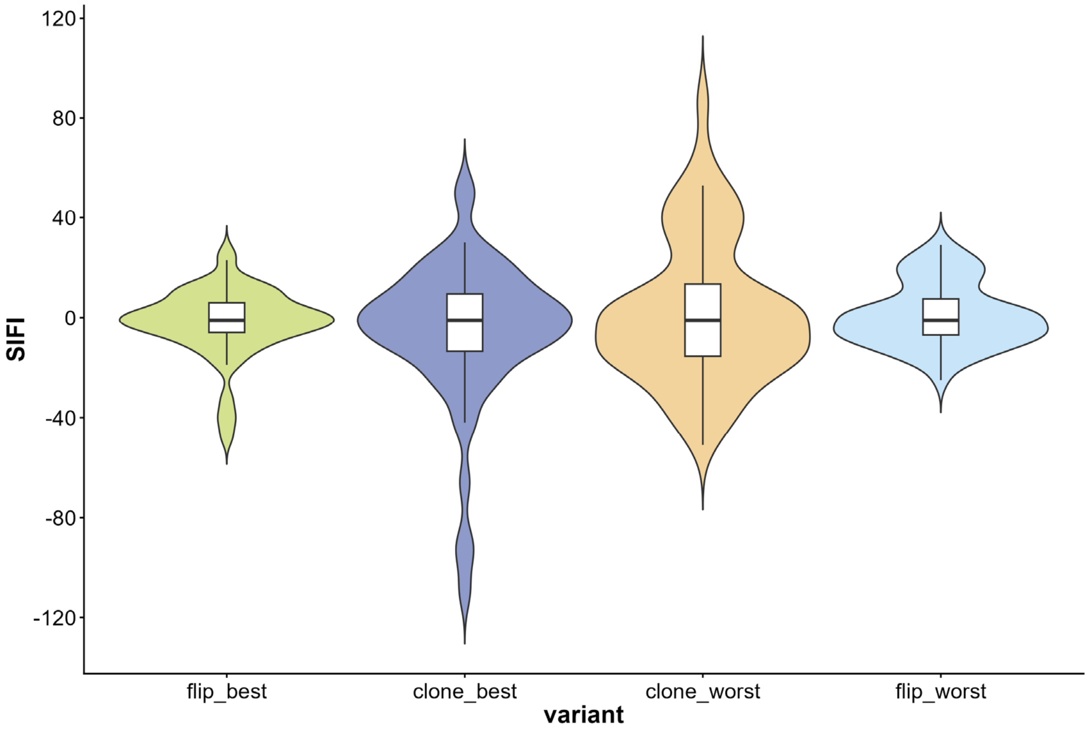

Figure S5 Violin plots with embedded box plots comparing SIFI values calculated using four methods

SIFI, survival-inferred fragility index.

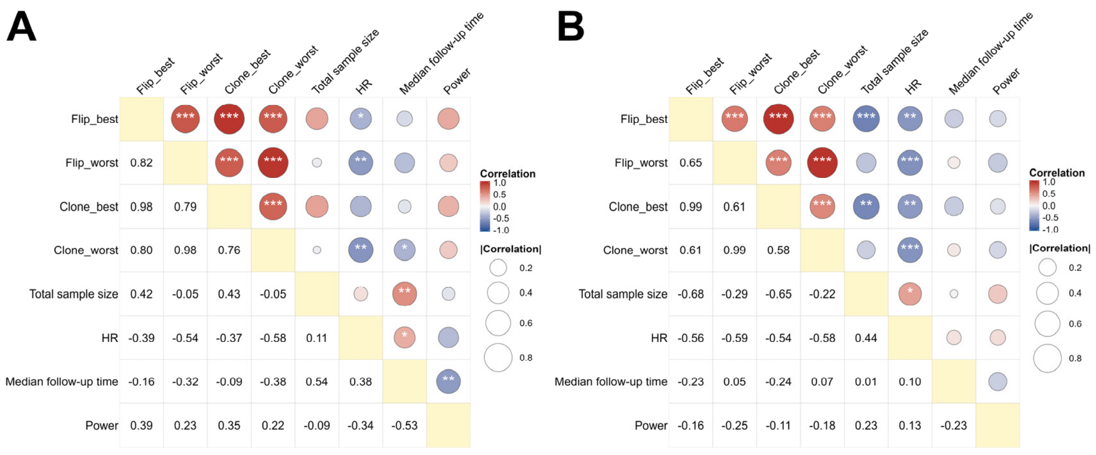

Figure S6 Correlation heatmap between SIFI and trial parameters in all included colorectal cancer pairwise comparisons. (A) Positive results, (B) Negative results. Asterisks indicate statistical significance: *$P < 0.05$, **$P < 0.01$, ***$P < 0.001$.

SIFI, survival-inferred fragility index

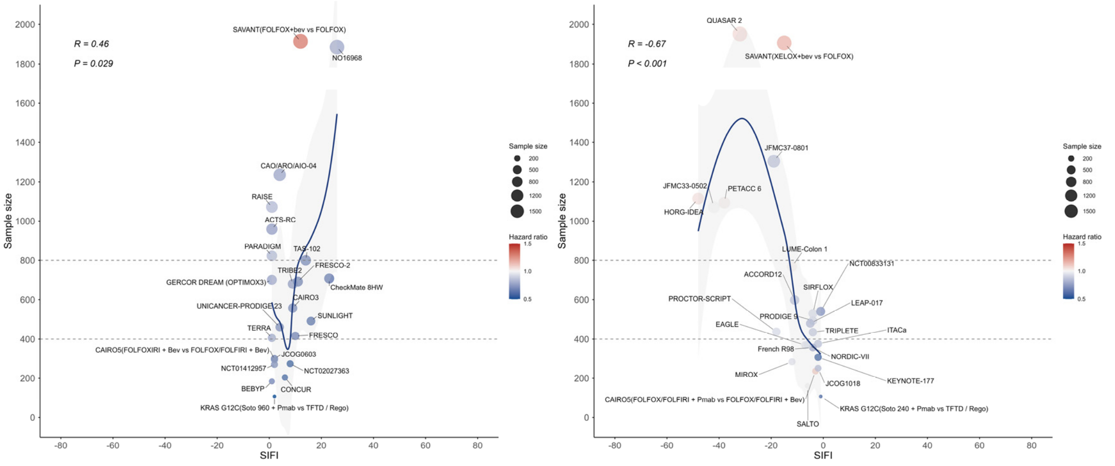

Figure S7 Correlation between SIFI and sample size after excluding three-arm trials. (A) Positive results, (B) Negative results.

Each point represents an individual trial, with point size proportional to sample size and color indicating the hazard ratio. The solid blue line represents the LOESS fit, and the horizontal dashed line indicates the conventional significance threshold. SIFI, survival-inferred fragility index; LOESS, locally estimated scatterplot smoothing.

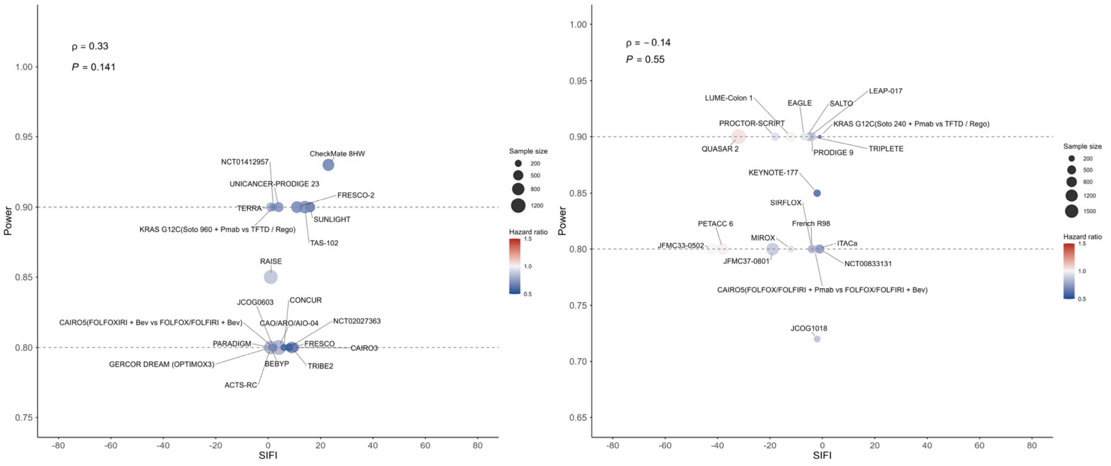

Figure S8 Correlation analysis between SIFI and statistical power after excluding three-arm trials. (A) Positive results, (B) Negative results.

SIFI, survival-inferred fragility index

References

1. Andre, T.; Elez, E.; Lenz, H.J.; Jensen, L.H.; Touchefeu, Y.; Van Cutsem, E.; Garcia-Carbonero, R.; Tougeron, D.; Mendez, G.A.; Schenker, M.; et al. Nivolumab plus ipilimumab versus nivolumab in microsatellite instability-high metastatic colorectal cancer (CheckMate 8HW): a randomised, open-label, phase 3 trial. *Lancet.* **2025**, *405*, 383–395.
2. Yoshino, T.; Andre, T.; Kim, T.W.; Yong, W.P.; Shiu, K.K.; Jensen, B.V.; Jensen, L.H.; Punt, C.J.A.; Smith, D.; Garcia-Carbonero, R.; et al. Pembrolizumab in Asian patients with microsatellite-instability-high/mismatch-repair-deficient colorectal cancer. *Cancer Sci.* **2023**, *114*, 1026–1036.
3. Andre, T.; Vernerey, D.; Im, S.A.; Bodoky, G.; Buzzoni, R.; Reingold, S.; Rivera, F.; McKendrick, J.; Scheithauer, W.; Ravit, G.; et al. Bevacizumab as adjuvant treatment of colon cancer: updated results from the S-AVANT phase III study by the GERCOR Group. *Ann Oncol.* **2020**, *31*, 246–256.
4. Aparicio, T.; Ghiringhelli, F.; Boige, V.; Le Malicot, K.; Taieb, J.; Bouche, O.; Phelip, J.M.; Francois, E.; Borel, C.; Faroux, R.; et al. Bevacizumab Maintenance Versus No Maintenance During Chemotherapy-Free Intervals in Metastatic Colorectal Cancer: A Randomized Phase III Trial (PRODIGE 9). *J Clin Oncol.* **2018**, *36*, 674–681.
5. Oki, E.; Murata, A.; Yoshida, K.; Maeda, K.; Ikejiri, K.; Munemoto, Y.; Sasaki, K.; Matsuda, C.; Kotake, M.; Suenaga, T.; et al. A randomized phase III trial comparing S-1 versus UFT as adjuvant chemotherapy for stage II/III rectal cancer (JFMC35-C1: ACTS-RC). *Ann Oncol.* **2016**, *27*, 1266–1272.
6. Passardi, A.; Nanni, O.; Tassinari, D.; Turci, D.; Cavanna, L.; Fontana, A.; Ruscelli, S.; Mucciarini, C.; Lorusso, V.; Ragazzini, A.; et al. Effectiveness of bevacizumab added to standard chemotherapy in metastatic colorectal cancer: final results for first-line treatment from the ITACa randomized clinical trial. *Ann Oncol.* **2015**, *26*, 1201–1207.
7. Pietrantonio, F.; Salvatore, L.; Esaki, T.; Modest, D.P.; Lopez-Bravo, D.P.; Taieb, J.; Karamouzis, M.V.; Ruiz-Garcia, E.; Kim, T.W.; Kuboki, Y.; et al. Overall Survival Analysis of the Phase III CodeBreaK 300 Study of Sotorasib Plus Panitumumab Versus Investigator's Choice in Chemorefractory KRAS G12C Colorectal Cancer. *J Clin Oncol.* **2025**, *43*, 2147–2154.
8. Prager, G.W.; Taieb, J.; Fakih, M.; Ciardiello, F.; Van Cutsem, E.; Elez, E.; Cruz, F.M.; Wyrwicz, L.; Stroyakovskiy, D.; Papai, Z.; et al. Trifluridine-Tipiracil and Bevacizumab in Refractory Metastatic Colorectal Cancer. *N Engl J Med.* **2023**, *388*, 1657–1667.
9. Rodel, C.; Graeven, U.; Fietkau, R.; Hohenberger, W.; Hothorn, T.; Arnold, D.; Hofheinz, R.D.; Ghadimi, M.; Wolff, H.A.; Lang-Welzenbach, M.; et al. Oxaliplatin added to fluorouracil-based preoperative chemoradiotherapy and postoperative chemotherapy of locally advanced rectal cancer (the German CAO/ARO/AIO-04 study): final results of the multicentre, open-label, randomised, phase 3 trial. *Lancet Oncol.* **2015**, *16*, 979–989.
10. Rossini, D.; Antoniotti, C.; Lonardi, S.; Pietrantonio, F.; Moretto, R.; Antonuzzo, L.;

Boccaccino, A.; Morano, F.; Brugia, M.; Pozzo, C.; et al. Upfront Modified Fluorouracil, Leucovorin, Oxaliplatin, and Irinotecan Plus Panitumumab Versus Fluorouracil, Leucovorin, and Oxaliplatin Plus Panitumumab for Patients With RAS/BRAF Wild-Type Metastatic Colorectal Cancer: The Phase III TRIPLETE Study by GONO. *J Clin Oncol.* **2022**, *40*, 2878–2888.
11. Sadahiro, S.; Tsuchiya, T.; Sasaki, K.; Kondo, K.; Katsumata, K.; Nishimura, G.; Kakeji, Y.; Baba, H.; Sato, S.; Koda, K.; et al. Randomized phase III trial of treatment duration for oral uracil and tegafur plus leucovorin as adjuvant chemotherapy for patients with stage IIB/III colon cancer: final results of JFMC33-0502. *Ann Oncol.* **2015**, *26*, 2274–2280.
12. Schmoll, H.J.; Stein, A.; Van Cutsem, E.; Price, T.; Hofheinz, R.D.; Nordlinger, B.; Daisne, J.F.; Janssens, J.; Brenner, B.; Reinel, H.; et al. Pre- and Postoperative Capecitabine Without or With Oxaliplatin in Locally Advanced Rectal Cancer: PETACC 6 Trial by EORTC GITCG and ROG, AIO, AGITG, BGDO, and FFCD. *J Clin Oncol.* **2021**, *39*, 17–29.
13. Schmoll, H.J.; Tabernero, J.; Maroun, J.; de Braud, F.; Price, T.; Van Cutsem, E.; Hill, M.; Hoersch, S.; Rittweger, K.; Haller, D.G. Capecitabine Plus Oxaliplatin Compared With Fluorouracil/Folinic Acid As Adjuvant Therapy for Stage III Colon Cancer: Final Results of the NO16968 Randomized Controlled Phase III Trial. *J Clin Oncol.* **2015**, *33*, 3733–3740.
14. Simkens, L.H.; van Tinteren, H.; May, A.; ten Tije, A.J.; Creemers, G.J.; Loosveld, O.J.; de Jongh, F.E.; Erdkamp, F.L.; Erjavec, Z.; van der Torren, A.M.; et al. Maintenance treatment with capecitabine and bevacizumab in metastatic colorectal cancer (CAIRO3): a phase 3 randomised controlled trial of the Dutch Colorectal Cancer Group. *Lancet.* **2015**, *385*, 1843–1852.
15. Souglakos, J.; Boukovinas, I.; Kakolyris, S.; Xynogalos, S.; Ziras, N.; Athanasiadis, A.; Androulakis, N.; Christopoulou, A.; Vaslamatzis, M.; Ardavanis, A.; et al. Three- versus six-month adjuvant FOLFOX or CAPOX for high-risk stage II and stage III colon cancer patients: the efficacy results of Hellenic Oncology Research Group (HORG) participation to the International Duration Evaluation of Adjuvant Chemotherapy (IDEA) project. *Ann Oncol.* **2019**, *30*, 1304–1310.
16. Tabernero, J.; Grothey, A.; Van Cutsem, E.; Yaeger, R.; Wasan, H.; Yoshino, T.; Desai, J.; Ciardiello, F.; Loupakis, F.; Hong, Y.S.; et al. Encorafenib Plus Cetuximab as a New Standard of Care for Previously Treated BRAF V600E-Mutant Metastatic Colorectal Cancer: Updated Survival Results and Subgroup Analyses from the BEACON Study. *J Clin Oncol.* **2021**, *39*, 273–284.
17. Tabernero, J.; Yoshino, T.; Cohn, A.L.; Obermannova, R.; Bodoky, G.; Garcia-Carbonero, R.; Ciuleanu, T.E.; Portnoy, D.C.; Van Cutsem, E.; Grothey, A.; et al. Ramucirumab versus placebo in combination with second-line FOLFIRI in patients with metastatic colorectal carcinoma that progressed during or after first-line therapy with bevacizumab, oxaliplatin, and a fluoropyrimidine (RAISE): a randomised, double-blind, multicentre, phase 3 study. *Lancet Oncol.* **2015**, *16*, 499–508.
18. Takashima, A.; Garcia-Alfonso, P.; Manneh, R.; Besen, A.A.; Hong, Y.S.; Cuyle, P.J.; Yanez, P.; Burge, M.; Yoshino, T.; Kim, T.W.; et al. Olaparib with or without

bevacizumab versus bevacizumab plus a fluoropyrimidine as maintenance therapy in advanced colorectal cancer: The randomized phase 3 LYNK-003 study. *Eur J Cancer.* **2024**, *205*, 114036.

19. Tomita, N.; Kunieda, K.; Maeda, A.; Hamada, C.; Yamanaka, T.; Sato, T.; Yoshida, K.; Boku, N.; Nezu, R.; Yamaguchi, S.; et al. Phase III randomised trial comparing 6 vs. 12-month of capecitabine as adjuvant chemotherapy for patients with stage III colon cancer: final results of the JFMC37-0801 study. *Br J Cancer.* **2019**, *120*, 689–696.
20. Tournigand, C.; Chibaudel, B.; Samson, B.; Scheithauer, W.; Vernerey, D.; Mesange, P.; Lledo, G.; Viret, F.; Ramee, J.F.; Tubiana-Mathieu, N.; et al. Bevacizumab with or without erlotinib as maintenance therapy in patients with metastatic colorectal cancer (GERCOR DREAM; OPTIMOX3): a randomised, open-label, phase 3 trial. *Lancet Oncol.* **2015**, *16*, 1493–1505.
21. Van Cutsem, E.; Yoshino, T.; Lenz, H.J.; Lonardi, S.; Falcone, A.; Limon, M.L.; Saunders, M.; Sobrero, A.; Park, Y.S.; Ferreiro, R.; et al. Nintedanib for the treatment of patients with refractory metastatic colorectal cancer (LUME-Colon 1): a phase III, international, randomized, placebo-controlled study. *Ann Oncol.* **2018**, *29*, 1955–1963.
22. van Hazel, G.A.; Heinemann, V.; Sharma, N.K.; Findlay, M.P.; Ricke, J.; Peeters, M.; Perez, D.; Robinson, B.A.; Strickland, A.H.; Ferguson, T.; et al. SIRFLOX: Randomized Phase III Trial Comparing First-Line mFOLFOX6 (Plus or Minus Bevacizumab) Versus mFOLFOX6 (Plus or Minus Bevacizumab) Plus Selective Internal Radiation Therapy in Patients With Metastatic Colorectal Cancer. *J Clin Oncol.* **2016**, *34*, 1723–1731.
23. Watanabe, J.; Muro, K.; Shitara, K.; Yamazaki, K.; Shiozawa, M.; Ohori, H.; Takashima, A.; Yokota, M.; Makiyama, A.; Akazawa, N.; et al. Panitumumab vs Bevacizumab Added to Standard First-line Chemotherapy and Overall Survival Among Patients With RAS Wild-type, Left-Sided Metastatic Colorectal Cancer: A Randomized Clinical Trial. *JAMA.* **2023**, *329*, 1271–1282.
24. Xu, J.; Kim, T.W.; Shen, L.; Sriuranpong, V.; Pan, H.; Xu, R.; Guo, W.; Han, S.W.; Liu, T.; Park, Y.S.; et al. Results of a Randomized, Double-Blind, Placebo-Controlled, Phase III Trial of Trifluridine/Tipiracil (TAS-102) Monotherapy in Asian Patients With Previously Treated Metastatic Colorectal Cancer: The TERRA Study. *J Clin Oncol.* **2018**, *36*, 350–358.
25. Breugom, A.J.; van Gijn, W.; Muller, E.W.; Berglund, A.; van den Broek, C.B.M.; Fokstuen, T.; Gelderblom, H.; Kapiteijn, E.; Leer, J.W.H.; Marijnen, C.A.M.; et al. Adjuvant chemotherapy for rectal cancer patients treated with preoperative (chemo)radiotherapy and total mesorectal excision: a Dutch Colorectal Cancer Group (DCCG) randomized phase III trial. *Ann Oncol.* **2015**, *26*, 696–701.
26. Bond, M.J.G.; Bolhuis, K.; Loosveld, O.J.L.; de Groot, J.W.B.; Droogendijk, H.; Helgason, H.H.; Hendriks, M.P.; Klaase, J.M.; Kazemier, G.; Liem, M.S.L.; et al. First-line systemic treatment strategies in patients with initially unresectable colorectal cancer liver metastases (CAIRO5): an open-label, multicentre, randomised, controlled, phase 3 study from the Dutch Colorectal Cancer Group. *Lancet Oncol.*

2023, *24*, 757–771.
27. Azria, D.; Doyen, J.; Jarlier, M.; Martel-Lafay, I.; Hennequin, C.; Etienne, P.; Vendrely, V.; Francois, E.; de La Roche, G.; Bouche, O.; et al. Late toxicities and clinical outcome at 5 years of the ACCORD 12/0405-PRODIGE 02 trial comparing two neoadjuvant chemoradiotherapy regimens for intermediate-risk rectal cancer. *Ann Oncol.* **2017**, *28*, 2436–2442.
28. Kanemitsu, Y.; Shimizu, Y.; Mizusawa, J.; Inaba, Y.; Hamaguchi, T.; Shida, D.; Ohue, M.; Komori, K.; Shiomi, A.; Shiozawa, M.; et al. Hepatectomy Followed by mFOLFOX6 Versus Hepatectomy Alone for Liver-Only Metastatic Colorectal Cancer (JCOG0603): A Phase II or III Randomized Controlled Trial. *J Clin Oncol.* **2021**, *39*, 3789–3799.
29. Takashima, A.; Hamaguchi, T.; Mizusawa, J.; Nagashima, F.; Ando, M.; Ojima, H.; Denda, T.; Watanabe, J.; Shinozaki, K.; Baba, H.; et al. Oxaliplatin Added to Fluoropyrimidine/Bevacizumab as Initial Therapy for Unresectable Metastatic Colorectal Cancer in Older Patients: A Multicenter, Randomized, Open-Label Phase III Trial (JCOG1018). *J Clin Oncol.* **2024**, *42*, 3967–3976.
30. Zhang, J.; Chi, P.; Shi, L.; Cui, L.; Gao, J.; Li, W.; Wei, H.; Cheng, L.; Huang, Z.; Cai, G.; et al. Neoadjuvant Modified Infusional Fluorouracil, Leucovorin, and Oxaliplatin With or Without Radiation Versus Fluorouracil Plus Radiation for Locally Advanced Rectal Cancer: Updated Results of the FOWARC Study After a Median Follow-Up of 10 Years. *J Clin Oncol.* **2025**, *43*, 633–640.
31. Bujko, K.; Wyrwicz, L.; Rutkowski, A.; Malinowska, M.; Pietrzak, L.; Krynski, J.; Michalski, W.; Oledzki, J.; Kusnierz, J.; Zajac, L.; et al. Long-course oxaliplatin-based preoperative chemoradiation versus 5 x 5 Gy and consolidation chemotherapy for cT4 or fixed cT3 rectal cancer: results of a randomized phase III study. *Ann Oncol.* **2016**, *27*, 834–842.
32. Conroy, T.; Bosset, J.F.; Etienne, P.L.; Rio, E.; Francois, E.; Mesgouez-Nebout, N.; Vendrely, V.; Artignan, X.; Bouche, O.; Gargot, D.; et al. Neoadjuvant chemotherapy with FOLFIRINOX and preoperative chemoradiotherapy for patients with locally advanced rectal cancer (UNICANCER-PRODIGE 23): a multicentre, randomised, open-label, phase 3 trial. *Lancet Oncol.* **2021**, *22*, 702–715.
33. Cremolini, C.; Antoniotti, C.; Rossini, D.; Lonardi, S.; Loupakis, F.; Pietrantonio, F.; Bordonaro, R.; Latiano, T.P.; Tamburini, E.; Santini, D.; et al. Upfront FOLFOXIRI plus bevacizumab and reintroduction after progression versus mFOLFOX6 plus bevacizumab followed by FOLFIRI plus bevacizumab in the treatment of patients with metastatic colorectal cancer (TRIBE2): a multicentre, open-label, phase 3, randomised, controlled trial. *Lancet Oncol.* **2020**, *21*, 497–507.
34. Delbaldo, C.; Ychou, M.; Zawadi, A.; Douillard, J.Y.; Andre, T.; Guerin-Meyer, V.; Rougier, P.; Dupuis, O.; Faroux, R.; Jouhaud, A.; et al. Postoperative irinotecan in resected stage II-III rectal cancer: final analysis of the French R98 Intergroup trialdagger. *Ann Oncol.* **2015**, *26*, 1208–1215.
35. Dasari, A.; Lonardi, S.; Garcia-Carbonero, R.; Elez, E.; Yoshino, T.; Sobrero, A.; Yao, J.; Garcia-Alfonso, P.; Kocsis, J.; Cubillo Gracian, A.; et al. Fruquintinib versus placebo in patients with refractory metastatic colorectal cancer (FRESCO-2): an

international, multicentre, randomised, double-blind, phase 3 study. *Lancet.* **2023**, *402*, 41–53.
36. Eng, C.; Kim, T.W.; Bendell, J.; Argiles, G.; Tebbutt, N.C.; Di Bartolomeo, M.; Falcone, A.; Fakih, M.; Kozloff, M.; Segal, N.H.; et al. Atezolizumab with or without cobimetinib versus regorafenib in previously treated metastatic colorectal cancer (IMblaze370): a multicentre, open-label, phase 3, randomised, controlled trial. *Lancet Oncol.* **2019**, *20*, 849–861.
37. Fakih, M.G.; Salvatore, L.; Esaki, T.; Modest, D.P.; Lopez-Bravo, D.P.; Taieb, J.; Karamouzis, M.V.; Ruiz-Garcia, E.; Kim, T.W.; Kuboki, Y.; et al. Sotorasib plus Panitumumab in Refractory Colorectal Cancer with Mutated KRAS G12C. *N Engl J Med.* **2023**, *389*, 2125–2139.
38. Guren, T.K.; Thomsen, M.; Kure, E.H.; Sorbye, H.; Glimelius, B.; Pfeiffer, P.; Osterlund, P.; Sigurdsson, F.; Lothe, I.M.B.; Dalsgaard, A.M.; et al. Cetuximab in treatment of metastatic colorectal cancer: final survival analyses and extended RAS data from the NORDIC-VII study. *Br J Cancer.* **2017**, *116*, 1271–1278.
39. Hebbar, M.; Chibaudel, B.; Andre, T.; Mineur, L.; Smith, D.; Louvet, C.; Dutel, J.L.; Ychou, M.; Legoux, J.L.; Mabro, M.; et al. FOLFOX4 versus sequential dose-dense FOLFOX7 followed by FOLFIRI in patients with resectable metastatic colorectal cancer (MIROX): a pragmatic approach to chemotherapy timing with perioperative or postoperative chemotherapy from an open-label, randomized phase III trial. *Ann Oncol.* **2015**, *26*, 1040.
40. Iwamoto, S.; Takahashi, T.; Tamagawa, H.; Nakamura, M.; Munemoto, Y.; Kato, T.; Hata, T.; Denda, T.; Morita, Y.; Inukai, M.; et al. FOLFIRI plus bevacizumab as second-line therapy in patients with metastatic colorectal cancer after first-line bevacizumab plus oxaliplatin-based therapy: the randomized phase III EAGLE study. *Ann Oncol.* **2015**, *26*, 1427–1433.
41. Kawazoe, A.; Xu, R.H.; Garcia-Alfonso, P.; Passhak, M.; Teng, H.W.; Shergill, A.; Gumus, M.; Qvortrup, C.; Stintzing, S.; Towns, K.; et al. Lenvatinib Plus Pembrolizumab Versus Standard of Care for Previously Treated Metastatic Colorectal Cancer: Final Analysis of the Randomized, Open-Label, Phase III LEAP-017 Study. *J Clin Oncol.* **2024**, *42*, 2918–2927.
42. Kerr, R.S.; Love, S.; Segelov, E.; Johnstone, E.; Falcon, B.; Hewett, P.; Weaver, A.; Church, D.; Scudder, C.; Pearson, S.; et al. Adjuvant capecitabine plus bevacizumab versus capecitabine alone in patients with colorectal cancer (QUASAR 2): an open-label, randomised phase 3 trial. *Lancet Oncol.* **2016**, *17*, 1543–1557.
43. Kim, T.W.; Elme, A.; Kusic, Z.; Park, J.O.; Udrea, A.A.; Kim, S.Y.; Ahn, J.B.; Valencia, R.V.; Krishnan, S.; Bilic, A.; et al. A phase 3 trial evaluating panitumumab plus best supportive care vs best supportive care in chemorefractory wild-type KRAS or RAS metastatic colorectal cancer. *Br J Cancer.* **2016**, *115*, 1206–1214.
44. Kopetz, S.; Grothey, A.; Yaeger, R.; Van Cutsem, E.; Desai, J.; Yoshino, T.; Wasan, H.; Ciardiello, F.; Loupakis, F.; Hong, Y.S.; et al. Encorafenib, Binimetinib, and Cetuximab in BRAF V600E-Mutated Colorectal Cancer. *N Engl J Med.* **2019**, *381*, 1632–1643.
45. Kwakman, J.J.M.; Simkens, L.H.J.; van Rooijen, J.M.; van de Wouw, A.J.; Ten Tije,

A.J.; Creemers, G.J.M.; Hendriks, M.P.; Los, M.; van Alphen, R.J.; Polee, M.B.; et al. Randomized phase III trial of S-1 versus capecitabine in the first-line treatment of metastatic colorectal cancer: SALTO study by the Dutch Colorectal Cancer Group. *Ann Oncol.* **2017**, *28*, 1288–1293.

46. Li, J.; Qin, S.; Xu, R.; Yau, T.C.; Ma, B.; Pan, H.; Xu, J.; Bai, Y.; Chi, Y.; Wang, L.; et al. Regorafenib plus best supportive care versus placebo plus best supportive care in Asian patients with previously treated metastatic colorectal cancer (CONCUR): a randomised, double-blind, placebo-controlled, phase 3 trial. *Lancet Oncol.* **2015**, *16*, 619–629.

47. Li, J.; Qin, S.; Xu, R.H.; Shen, L.; Xu, J.; Bai, Y.; Yang, L.; Deng, Y.; Chen, Z.D.; Zhong, H.; et al. Effect of Fruquintinib vs Placebo on Overall Survival in Patients With Previously Treated Metastatic Colorectal Cancer: The FRESCO Randomized Clinical Trial. *JAMA.* **2018**, *319*, 2486–2496.

48. Luo, H.Y.; Li, Y.H.; Wang, W.; Wang, Z.Q.; Yuan, X.; Ma, D.; Wang, F.H.; Zhang, D.S.; Lin, D.R.; Lin, Y.C.; et al. Single-agent capecitabine as maintenance therapy after induction of XELOX (or FOLFOX) in first-line treatment of metastatic colorectal cancer: randomized clinical trial of efficacy and safety. *Ann Oncol.* **2016**, *27*, 1074–1081.

49. Masi, G.; Salvatore, L.; Boni, L.; Loupakis, F.; Cremolini, C.; Fornaro, L.; Schirripa, M.; Cupini, S.; Barbara, C.; Safina, V.; et al. Continuation or reintroduction of bevacizumab beyond progression to first-line therapy in metastatic colorectal cancer: final results of the randomized BEBYP trial. *Ann Oncol.* **2015**, *26*, 724–730.

50. Mayer, R.J.; Van Cutsem, E.; Falcone, A.; Yoshino, T.; Garcia-Carbonero, R.; Mizunuma, N.; Yamazaki, K.; Shimada, Y.; Tabernero, J.; Komatsu, Y.; et al. Randomized trial of TAS-102 for refractory metastatic colorectal cancer. *N Engl J Med.* **2015**, *372*, 1909–1919.
